# Microbiota identified from preserved *Anopheles*

**DOI:** 10.1186/s12936-021-03754-7

**Published:** 2021-05-22

**Authors:** Bianca E Silva, Zvifadzo Matsena Zingoni, Lizette L. Koekemoer, Yael L. Dahan-Moss

**Affiliations:** 1 Wits Research Institute for Malaria, School of Pathology, Faculty of Health Sciences, University of the Witwatersrand, Johannesburg, South Africa; 2 Centre for Emerging Zoonotic and Parasitic Diseases, National Institute for Communicable Diseases of the National Health Laboratory Service, Johannesburg, South Africa; 3 Division of Epidemiology and Biostatistics, School of Public Health, University of the Witwatersrand, Parktown, South Africa

**Keywords:** *Anopheles arabiensis*, *Anopheles funestus*, Culturomics, Next-generation sequencing, RNA*later*^®^, Silica

## Abstract

**Background:**

Mosquito species from the *Anopheles gambiae* complex and the *Anopheles funestus* group are dominant African malaria vectors. Mosquito microbiota play vital roles in physiology and vector competence. Recent research has focused on investigating the mosquito microbiota, especially in wild populations. Wild mosquitoes are preserved and transported to a laboratory for analyses. Thus far, microbial characterization post-preservation has been investigated in only *Aedes vexans* and *Culex pipiens*. Investigating the efficacy of cost-effective preservatives has also been limited to AllProtect reagent, ethanol and nucleic acid preservation buffer. This study characterized the microbiota of African *Anopheles* vectors: *Anopheles arabiensis* (member of the *An. gambiae* complex) and *An. funestus* (member of the *An. funestus* group), preserved on silica desiccant and RNA*later*^®^ solution.

**Methods:**

Microbial composition and diversity were characterized using culture-dependent (midgut dissections, culturomics, MALDI-TOF MS) and culture-independent techniques (abdominal dissections, DNA extraction, next-generation sequencing) from laboratory (colonized) and field-collected mosquitoes. Colonized mosquitoes were either fresh (non-preserved) or preserved for 4 and 12 weeks on silica or in RNA*later*^®^. Microbiota were also characterized from field-collected *An. arabiensis* preserved on silica for 8, 12 and 16 weeks.

**Results:**

*Elizabethkingia anophelis* and *Serratia oryzae* were common between both vector species, while *Enterobacter cloacae* and *Staphylococcus epidermidis* were specific to females and males, respectively. Microbial diversity was not influenced by sex, condition (fresh or preserved), preservative, or preservation time-period; however, the type of bacterial identification technique affected all microbial diversity indices.

**Conclusions:**

This study broadly characterized the microbiota of *An. arabiensis* and *An. funestus*. Silica- and RNA*later*^®^-preservation were appropriate when paired with culture-dependent and culture-independent techniques, respectively. These results broaden the selection of cost-effective methods available for handling vector samples for downstream microbial analyses.

**Supplementary Information:**

The online version contains supplementary material available at 10.1186/s12936-021-03754-7.

## Background

Malaria is a vector-borne disease that disproportionately affects the youth and pregnant women in underdeveloped countries [1]. In 2019, 94% of the global malaria cases were confined to the World Health Organization (WHO) African Region [1]. Malaria is caused by the *Plasmodium* parasite and is transmitted to humans by the bite of an infected female *Anopheles* mosquito. *Plasmodium falciparum* is the dominant malaria parasite in Africa and is transmitted by members of the *Anopheles gambiae* complex and the *Anopheles funestus* group [1]. Targeting vectors through novel interventions could reduce malaria transmission.

The mosquito’s midgut micro-organismal community has gained interest for its potential to reduce malaria transmission. Mosquitoes naturally acquire micro-organisms from their environment, which colonize in the midgut and form symbiotic relationships that contribute to mosquito physiology [2–13]. In *Anopheles*, microbiota contribute to digestion and nutrient attainment [14–18]; fertility, fecundity and behaviour [19–22]; insecticide resistance [23–27]; development and homeostasis [4, 28–35]; and vector immunity [2, 5, 7, 31, 36–67]. *Anopheles* microbiota can be investigated in a vector-specific manner, which could aid future studies on the vector-microbiota-pathogen relationship.

Culturomics, a culture-dependent technique that involves growing bacteria using nutrient media, is commonly used to characterize the mosquito’s midgut bacteria as it is fast, cost-effective and provides reliable data [68–71]. Morphologically distinct colonies are isolated and subjected to matrix-assisted laser desorption ionization-time of flight (MALDI-TOF) mass spectrometry (MS). MALDI-TOF MS identifies bacteria based on their proteome, where proteins are cleaved into peptides and their molecular masses are used to create peptide mass fingerprints (PMFs) (reviewed by [71]). The PMFs of unknown bacteria are compared with the PMFs of known bacteria in a database for taxonomic identification [71].

However, certain bacteria cannot grow on selective media, and species identification is limited to the local database installed on the MALDI-TOF MS system [72, 73]. Next-generation sequencing (NGS), a sensitive, culture-independent approach, addresses these downfalls (reviewed by [74]). NGS identifies bacteria based on their genome: conserved regions of the prokaryotic 16S ribosomal ribonucleic acid (rRNA) gene are used for amplification, and hypervariable regions of the gene are used to identify taxa [75–77]. Yet, NGS is vulnerable to bias and can be costly and time-consuming [74, 78–81].

Thus, neither approach is superior: they are complementary, and using both provides a dataset of overlapping bacteria, as reported in many mosquito microbial studies [8, 11, 17, 18, 48, 52, 53, 61, 68, 82–85]. Both techniques provide data on microbial species composition and diversity, the latter of which can be measured using species richness, relative abundance, and species distribution [86, 87]. Common indices used to estimate diversity include the Shannon–Wiener index for species diversity, Simpson’s reciprocal index for relative abundance, and Pielou’s evenness index for species distribution [2, 18, 53, 85, 88].

As mosquito microbial studies are increasingly shifting to field-collected samples, field-caught mosquitoes are preserved and transported to a laboratory for analysis. This is because testing mosquitoes in field conditions is impractical due to the lack of a sterile environment and laboratory equipment. Field sites are also often far from suitably equipped laboratories. Although, the type of preservation method used is dependent on the type of downstream analysis being performed as certain preservatives are better suited for identifying specific entomological indicators [89].

Common preservation methods include fixation in reagents such as Allprotect Tissue Reagent, Carnoy’s solution (6:3:1 ethanol: chloroform: glacial acetic acid, with ferric chloride), ethanol (95%), nucleic acid preservation (NAP) buffer (ethylenediaminetetraacetic acid (EDTA), sodium citrate trisodium salt dihydrate, ammonium sulfate), or RNA*later*^®^; desiccation in drierite (anhydrous calcium sulfate) or silica; refrigeration at 4 °C or − 20 °C; and, cryopreservation in liquid nitrogen [89–94].

The microbiota of *Aedes vexans* and *Culex pipiens* have been identified post-preservation from AllProtect reagent, ethanol, and NAP buffer [93]. However, the efficacy of other commonly used, cost-effective preservatives, such as silica and RNA*later*^®^, has not been investigated. Silica preserves large quantities of specimens and ensures long-term preservation at room temperature [89, 92, 95], while RNA*later*^®^ preserves high-quality DNA and RNA and is most suitable for determining internal muscular anatomy [89]. Furthermore, the microbiota of preserved African *Anopheles* vectors has not been investigated.

Accordingly, this study assessed if the microbiota of African *Anopheles* vectors could be identified post-preservation from silica and RNA*later*^®^ using culture-dependent and culture-independent techniques. The microbiota of laboratory (colonized) *Anopheles arabiensis* (member of the *An. gambiae* complex) and *An. funestus* (member of the *An. funestus* group) were screened after preserving mosquitoes for up to 12 weeks with each preservative. Additionally, the microbiota of preserved field-collected *An. arabiensis* were characterized.

## Methods

### Biological material

Colonized mosquitoes were obtained from the Botha de Meillon Insectary, National Institute for Communicable Diseases (NICD), Johannesburg, South Africa. Two *Anopheles* species were used in this study: *An. arabiensis* (MBN colony) and *An. funestus* (FUMOZ colony). The MBN colony has mosquitoes from Mamfene, KwaZulu-Natal, South Africa, while the FUMOZ colony has mosquitoes from southern Mozambique [96, 97]. Mosquitoes (20 female and 20 male per species per repeat; three biological repeats) were collected between 0- and 24-h post-emergence (here forth called fresh samples). Additionally, 1 ml of each species’ larval rearing water was collected (three biological repeats).

Field-caught *An. arabiensis* were collected between June and August 2019 from Mamfene. These samples had been preserved on silica in microcentrifuge tubes and were retrieved from the departmental archive. Species and *Plasmodium*-infection status were confirmed using multiplex polymerase chain reaction (PCR) [98–100] and enzyme-linked immunosorbent assay (ELISA) [101], respectively, as part of a departmental Sterile Insect Technique (SIT) project. Samples were retrieved after 8, 12 and 16 weeks of preservation.

As mosquito density varies per season, field-collected mosquitoes were low in numbers because June, July and August are the winter months in South Africa (collection numbers are highest in summer and lowest in winter [102]). Mosquito density may have also been exacerbated by the extensive drought in South Africa, which probably left mosquitoes without local breeding pools ([103]: filter between June and August 2019 to view the lack of rainfall). Therefore, only four females were retrieved per time-period, and bacterial identification was performed using culturomics due to the low cost for the limited number of samples. Additionally, as this sample size was low, it was not used to represent the wild mosquito population, and comparisons between field-collected and colonized mosquitoes could not be made.

### Mosquito preservation

**Fig. 1:**
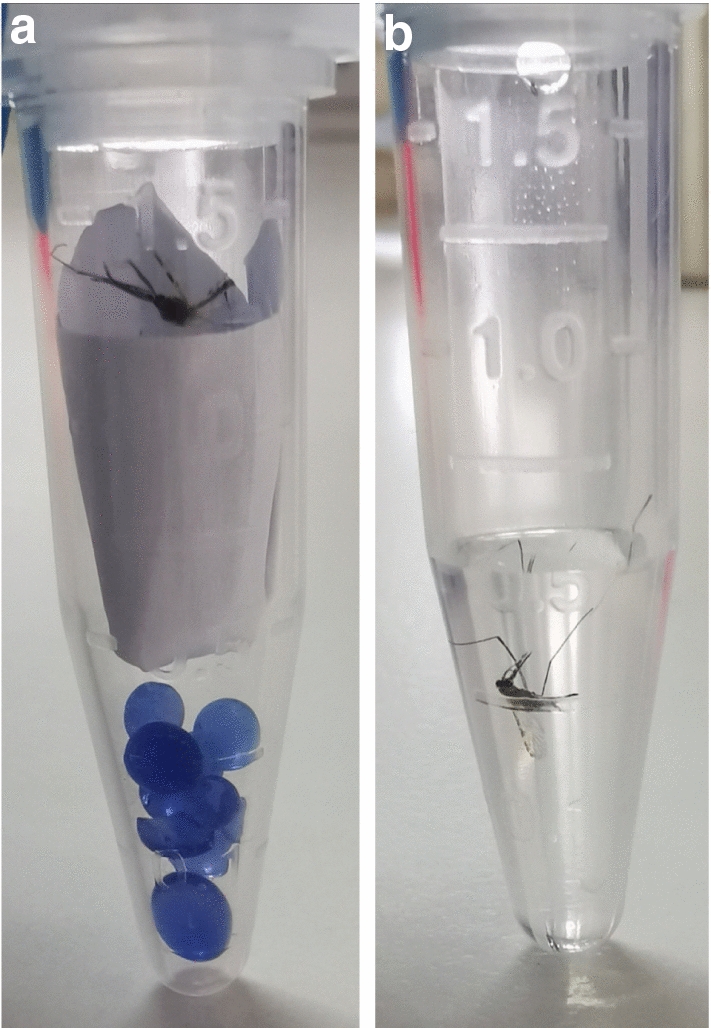
Mosquitoes preserved on **a** silica and in **b** RNA*later*^®^

Colonized mosquitoes were preserved on silica or in RNA*later*^®^ (20 females and 20 males per species, repeat, preservative, and preservation time-period; three biological repeats). Mosquitoes were preserved for 4 and 12 weeks per preservative. Prior to preservation, mosquitoes were immobilized at − 20 °C for 2 min. For silica preservation, mosquitoes were placed individually in 1.5-ml microcentrifuge tubes containing approximately five silica beads (silica gel blue self-indicator (copper sulphate-based), B&M Scientific, South Africa; cat no. CSGB0002) and were separated from silica using a piece of paper (Fig. 1a).

For RNA*later*^®^ preservation, mosquitoes were surface sterilized in 70% ethanol (v/v), left to dry at room temperature on a sterile piece of Kimwipe^®^ (Kimberly-Clark, TX, USA; cat no. 34155), and individually submerged in 0.5 ml of RNA*later*^®^ solution (Qiagen, Germany; cat no. 76106) in 1.5-ml microcentrifuge tubes (Fig. 1b). Samples preserved in RNA*later*^®^ were stored according to the manufacturer’s instructions (samples preserved for 4 weeks were stored at 4 °C, while samples preserved for 12 weeks were placed in RNA*later*^®^ overnight at 4 °C and subsequently stored at − 80 °C). For each species, a set of fresh (non-preserved) samples was collected for comparison. All supplies (silica beads, microcentrifuge tubes, separating paper, etc.) were sterilized prior to use, and swabs of these supplies were cultured to ensure they were not contaminated.

### Midgut dissection

Midgut dissections of fresh samples were performed aseptically per protocol by the WHO [104], where 4 µl of phosphate-buffered saline (PBS) was used per midgut; 20 midguts were pooled per species, sex, preservative, preservation time-period, and repeat.

### Abdominal dissections of preserved samples

Due to desiccation, mosquito abdomens were shrivelled and brittle. Therefore, mosquitoes were surface sterilized twice in 70% ethanol (v/v), and abdominal segments I to V were dissected and placed in sterile microcentrifuge tubes containing 4 µl of PBS; 20 abdominal segments were pooled per sex, species, preservative, preservation time-period, and repeat. Abdominal segments were then homogenized using a TissueLyser II, followed by centrifugation. Negative controls were set up per group of samples using PBS were carried through during downstream analyses. Additionally, abdomens tore apart easily when mosquitoes were submerged in RNA*later*^®^ and midguts could not be isolated. Thus, mosquitoes were removed from solution and blotted on tissue paper to remove excess RNA*later*^®^, followed by the adapted dissection method used for silica-preserved samples.

### Culture-dependent bacterial identification

Each homogenate (10 µl) was plated on individual selective media agar plates (Table 1). Inoculates were plated and aerobically incubated for a minimum of 16 h at 37 °C. Plates without observable colonies were re-incubated for 16 h to account for slow-growing bacteria. Following incubation, isolates were distinguished morphologically, and distinct colonies were selected for repeated sub-culture by re-inoculation on fresh primary agar plates and incubation for a minimum of 16 h at 37 °C. Plates without observable colonies were re-incubated for 16 h to account for slow-growing bacteria. Negative controls were set up for each plate type, and during subsequent incubation periods, to ensure plates were not contaminated during incubation. Each plate type was also inoculated with PBS negative controls from dissections.

**Table 1:** Selective media used and the bacteria they select for

Type of media agar plate	Selective for
MacConkey agar (DMP/NICD, South Africa; cat no. DMPA0315)	Non-fastidious gram-negative enteric bacteria [105]
10% blood agar (DMP/NICD, South Africa; cat no. DMPA0115)	A variety of fastidious bacteria [106]
Blood agar with nalidixic acid and colistin (DMP/NICD, South Africa; cat no. DMPA0110)	Gram-positive bacteria [107]
Chapman’s agar (or mannitol salt agar) (DMP/NICD, South Africa; cat no. DMPA0316)	Gram-positive bacteria, specifically staphylococci [108]
Brain–Heart Infusion (BHI) agar (DMP/NICD, South Africa; cat no. DMPB0120)	A variety of fastidious bacteria [109]

### Mass spectrometry (MALDI-TOF MS)

Each colony was placed directly on an individual spot on a 96-spot reusable MALDI-TOF target plate (Bruker Daltonics, Wissembourg, France; cat no. 8280800). Each spot was covered with 1 μl of α-Cyano-4-hydroxycinnamic acid (HCCA) matrix (Bruker Daltonics, Wissembourg, France; cat no. 8255344) diluted in standard solvent (50% acetonitrile: 47.5% water: 2.5% trifluoroacetic acid, Sigma-Aldrich, Lyon, France; cat no. 19182). The matrix was allowed to dry at room temperature, and the target plate was placed in the MALDI Biotyper^®^ with benchtop microflex™ LT/SH mass spectrometer (Bruker Daltonics, Germany). A bacterial test standard (Bruker Protein Calibration Standard I, Bruker Daltonics, Wissembourg, France; cat no. 8255343) was used according to the manufacturer’s instructions to control for loading and matrix. Spectra were compared with the MBT 7854 MSP Library database installed on the computer (Bruker Daltonics, Wissembourg, France; ref no. 182903). An isolate was identified when spectra had a log score value ≥ 1.9 [81]. Every unidentified isolate was tested successively, where a portion of the same colony was placed on a new spot on the target plate and identified as described.

### Culture-independent bacterial identification

To supplement culturomics, midguts of fresh mosquitoes and abdomens of preserved mosquitoes (preserved on silica and in RNA*later*^®^ for 4 and 12 weeks) were dissected and pooled (20 female and 20 male per species, preservative, preservation time-period, and repeat; three biological repeats) in sterile PBS as described. Additionally, 1 ml of each species’ larval rearing water was collected (three biological repeats). Bacterial DNA was extracted using the QIAamp^®^ DNA Microbiome Kit according to the manufacturer’s instructions (Qiagen, Germany; cat no. 51704). Negative controls were set up during each stage of DNA extraction, and extraction was also performed on PBS negative controls from abdominal dissections. Prior to sequencing, DNA quality and purity were measured using the NanoDrop™ 2000c spectrophotometer (Thermo Scientific, MA, USA; cat no. ND-2000C). Due to the high cost of NGS and the high number of negative controls, only experimental samples were sequenced. Nonetheless, negative controls were assessed using the NanoDrop™ 2000c spectrophotometer and were cultured to ensure no contamination.

Samples were sent to Macrogen Europe (Amsterdam, The Netherlands) for 16S rRNA gene sequencing targeting the V3-V4 regions with universal primers, Bakt 341F and Bakt 805R [110]. The Illumina MiSeq system was used to perform paired-end sequencing, and the Fast Length Adjustment of SHort reads (FLASH version 1.2.11) program was used to assemble reads [111]. Pre-processing (denoising) and clustering of sequences were performed with the CD-HIT-OTU and rDnaTools programs [112, 113]. Diversity analyses and taxonomy assignments were performed with the Quantitative Insights Into Microbial Ecology (QIIME) program (see Additional file 1 for OTUs with taxonomy assignment) [114].

### MALDI-TOF MS Library analysis

According to the MALDI-TOF MS MBT 7854 MSP Library, several bacteria are genetically indistinguishable from one another. Thus, the matching hints section of the library was used to compare MALDI-TOF MS and NGS results (see Additional file 2). Accordingly, indistinguishable bacteria were grouped, and a single bacterium was used to represent indistinguishable bacteria (Table 2). Representative bacteria were chosen based on which indistinguishable bacteria were also present in NGS results.

**Table 2:** Genetically indistinguishable bacteria grouped in this study

Indistinguishable bacteria (MBT 7854 MSP Library)	Representative bacterium
*Aeromonas hydrophila* and *Aeromonas veronii*	*A. hydrophila*
*Corynebacterium propinquum* and *Corynebacterium pseudodiphtheriticum*	*C. pseudodiphtheriticum*
*Delftia acidovorans* and *Delftia tsuruhatensis*	*D. tsuruhatensis*
*Elizabethkingia anophelis*, *Elizabethkingia meningoseptica*, and *Elizabethkingia miricola*	E. anophelis*
*Escherichia coli* and *Escherichia fergusonii*	*E. fergusonii*
*Klebsiella oxytoca*, *Raoultella ornithinolytica*, *Raoultella planticola* and *Raoultella terrigena*	R. ornithinolytica**

A single bacterium was used to represent bacteria that were indistinguishable by the MBT 7854 MSP Library

^*^E. anophelis is misidentified as *E. meningoseptica* by MALDI-TOF MS [115]

^**^R. ornithinolytica is misidentified as *Klebsiella pneumonia* or *K. oxytoca* [116, 117]

### Data analyses, diversity indices and statistical analyses

For fresh and field-collected samples, results are presented as accumulative data across replicates. This is because sample sizes for these groups were lower than the sample sizes of preserved mosquitoes due to the inclusion of preservatives and preservation time-periods. There was also a contrast in microbial composition between fresh and preserved samples identified by culture-independent techniques, which may be attributed to potential contamination. Thus, when comparing group, for example, sex (female or male) irrespective of species (*An. arabiensis* or *An. funestus*), condition (fresh or preserved), preservative (silica or RNA*later*^®^), preservation time-period (4 weeks or 12 weeks) or technique (culture-dependent or culture-independent), only commonly recurring bacteria (bacteria that appeared in at least 50% of replicates in the groups being compared) were reported.

Species richness, bacterial diversity, relative abundance, and evenness were calculated. Species richness was measured as the number of operational taxonomic units (OTUs) per sample. Indices were reported per replicate, and the mean index per group was calculated (Additional file 3). Diversity was measured using the Shannon–Wiener diversity index (H): the higher the value of H, the more diverse the community [118]. To estimate relative abundance as a measure of species dominance, Simpson’s reciprocal index (1/D) was calculated. 1/D measures the probability that a randomly selected species is the dominant species, where a score of one indicates the community is dominated by a single species [119]. E was used to estimate evenness, which ranges from zero to one with zero signifying no evenness and one signifying complete evenness [120].

Statistical analyses were performed at a 95% confidence interval assuming a 5% level of significance using STATA/IC version 16.1. As the data were not normally distributed (as per Shapiro–Wilk tests), non-parametric statistical analyses were performed. Two-sample Wilcoxon rank-sum (Mann–Whitney) tests were used to determine if diversity indices differed between (i) fresh females and males; (ii) fresh *An. arabiensis* and *An. funestus*; (iii) fresh mosquitoes and mosquitoes preserved for 4 weeks; (iv) fresh mosquitoes and mosquitoes preserved for 12 weeks; (v) mosquitoes preserved for 4 weeks and mosquitoes preserved for 12 weeks; (vi) silica preservation and RNA*later*^®^ preservation; and, (vii) culture-dependent and culture-independent techniques.

## Results

### Microbial composition of colonized and field-collected *Anopheles arabiensis*

**Fig. 2:**
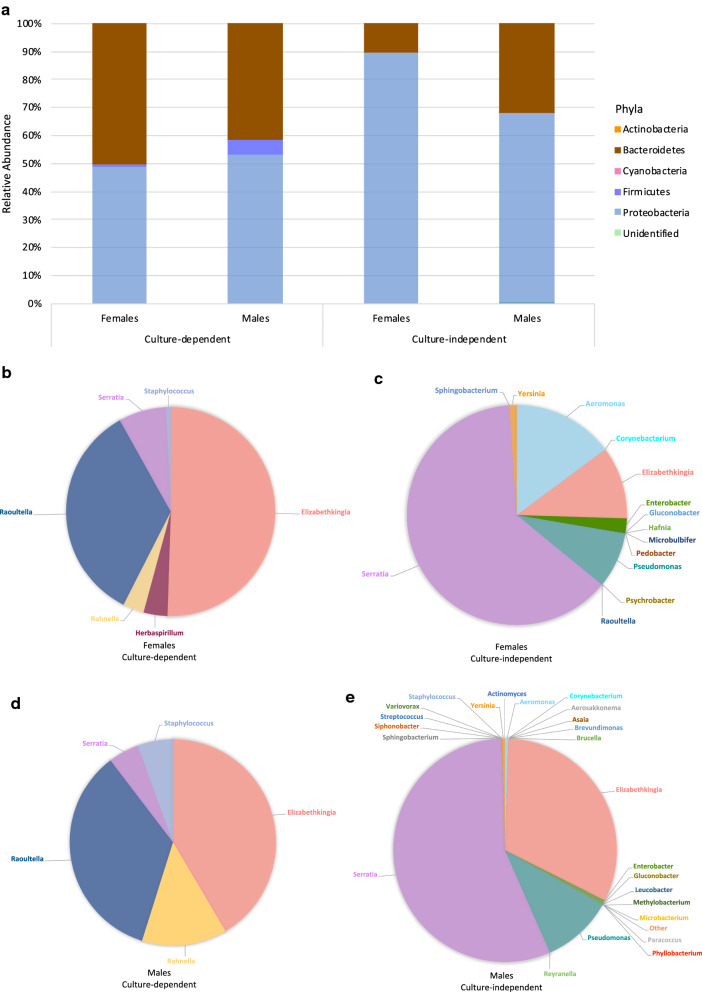
Relative abundance of *Anopheles arabiensis* microbial communities. Phyla are presented for **a** females and males, where each bar represents the average relative abundance identified either by culture-dependent or culture-independent techniques. Genera are presented for females identified by **b** culture-dependent and **c** culture-independent techniques and males identified by **d** culture-dependent and **e** culture-independent techniques

*Anopheles arabiensis* were predominantly colonized by Proteobacteria, irrespective of technique, followed by Bacteroidetes (Fig. 2a). While culture-dependent results estimated *Elizabethkingia* as the dominant genus in both *An. arabiensis* sexes, culture-independent results identified *Serratia* as the dominant genus (Fig. 2b–e).

Culture-dependent results identified *Serratia fonticola* as a commonly recurring bacterium, but *S. fonticola* was not identified by NGS. Thus, it is likely that *S. fonticola* identified by culturomics was actually *Serratia oryzae* because *S. oryzae* was identified as a commonly recurring bacterium by NGS and *S. oryzae* cannot be detected by the MBT 7854 MSP Library. Results of a pairwise alignment (performed using National Center for Biotechnology Information’s (NCBI) Basic Local Alignment Search Tool for nucleotide sequences (BLASTn) [121]: http://www.ncbi.nlm.nih.gov/BLAST/) of 16S rRNA sequences revealed 96.75% sequence similarity between *S. fonticola* (accession number: CP011254.1) and *S. oryzae* (accession number: NR_157762.1). Meanwhile, a pairwise alignment between *S. fonticola* and *Serratia liquefaciens* (accession number: UGYL01000001.1) and *S. fonticola* and *Serratia marcescens* (accession number: CP063354.1; the other *Serratia* species identified by NGS in this study), revealed 94.03% and 85.53% sequence similarity, respectively. Therefore, *S. oryzae* was used to represent *S. fonticola* throughout this study.

**Fig. 3:**
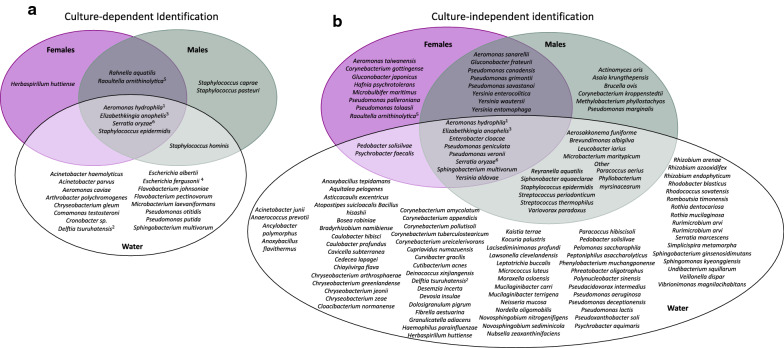
Bacteria identified by **a** culture-dependent and **b** culture-independent techniques from *Anopheles arabiensis* and the larval rearing water. Bacteria indistinguishable by MALDI-TOF MS include ^1^*A. hydrophila* and *A. veronii*; ^2^*D. acidovorans* and *D. tsuruhatensis*; ^3^*E. anophelis*, *E. meningoseptica*, and *E. miricola*; ^4^*E. coli* and *E. fergusonii*; ^5^* K. oxytoca*, *R. ornithinolytica*, *R. planticola*, and *R. terrigena*; and ^6^*S. fonticola* and *S. oryzae*

*Aeromonas hydrophila*, *E. anophelis* and *S. oryzae* were identified as common bacteria between female and male *An. arabiensis* and their larval rearing water, irrespective of identification technique (Fig. 3). *Staphylococcus epidermidis* was common between both sexes and the larval rearing water in culture-dependent results, but only in males and the larval rearing water in culture-independent results. *Raoultella ornithinolytica* was common between both sexes in culture-dependent results, but only in females in culture-independent results. Some bacteria were identified in a sex- and/or technique-dependent manner (see Additional file 4 for an overview of replicates).

Proteobacteria remained the dominant phylum in preserved *An. arabiensis* (Additional file 5). *Elizabethkingia anophelis* and *S. oryzae* were recurring bacteria that were common in silica-preserved and RNA*later*^®^-preserved *An. arabiensis* (Table 3; see Additional file 6 for replicate details). Irrespective of condition, *E. anophelis* and *S. oryzae* were recurring bacteria that were common between females and males. *Enterobacter cloacae* was specific to females and *S. epidermidis* was specific to males (Table 3; see Additional file 7 for replicate details). These sex-specific bacteria were also recurring bacteria in RNA*later*^®^-preserved (and not silica-preserved) samples. Overall, *E. anophelis* and *S. oryzae* were common between fresh and preserved female and male *An. arabiensis*.

**Table 3:** Recurring bacteria identified in *Anopheles arabiensis* according to preservatives and sex

Bacteria	Silica-preserved	RNA*later*^®^-preserved	Females	Males
*Asaia krungthepensis*		x		
*Cedecea lapagei*	x			
*Cutibacterium acnes*		x		
*Elizabethkingia anophelis*^*1*^	x	x	x	x
*Enterobacter cloacae*		x	x	
*Microbacterium maritypicum*		x		
*Moraxella osloensis*		x		
*Paracoccus aerius*		x		
*Phyllobacterium myrsinacearum*	x			
*Pseudomonas geniculata*	x			
*Pseudomonas veronii*	x			
*Raoultella ornithinolytica*	x			
*Serratia oryzae*^*2*^	x	x	x	x
*Staphylococcus epidermidis*		x		x
*Streptococcus thermophilus*		x		
*Yersinia aldovae*		x		

Recurring bacteria appeared in at least half of all replicates per group. Bacteria indistinguishable by MALDI-TOF MS include ^1^*E. anophelis*, *E. meningoseptica*, and *E. miricola*; and ^2^*S. fonticola* and *S. oryzae*

**Fig. 4:**
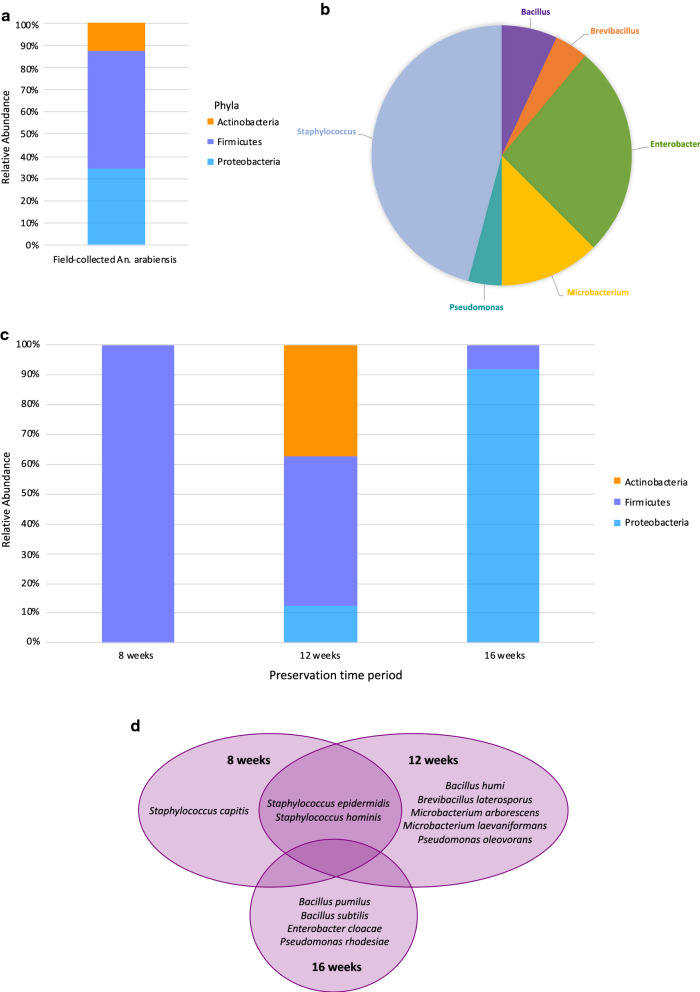
Microbial communities in preserved field-collected *Anopheles arabiensis*. Average relative abundance, irrespective of time-period, is presented according to **a** phyla and **b** genera. For each preservation time-period, the average relative abundance is presented according to **c** phyla per group, and microbial composition is presented according to **d** bacterial species per group

Field-collected *An. arabiensis* were predominantly colonized by Firmicutes (Fig. 4a). The dominant genus in field-collected *An. arabiensis* was *Staphylococcus* (Fig. 4b). Although Firmicutes was the dominant phylum in samples preserved for 8 and 12 weeks, samples preserved for 16 weeks were predominated by Proteobacteria (Fig. 4c). *Staphylococcus epidermidis* and *Staphylococcus hominis* were common between samples preserved for 8 and 12 weeks while samples preserved for 16 weeks did not have bacteria in common with samples preserved for 8 and 12 weeks (Fig. 4d).

### Microbial composition of colonized *Anopheles funestus*

**Fig. 5:**
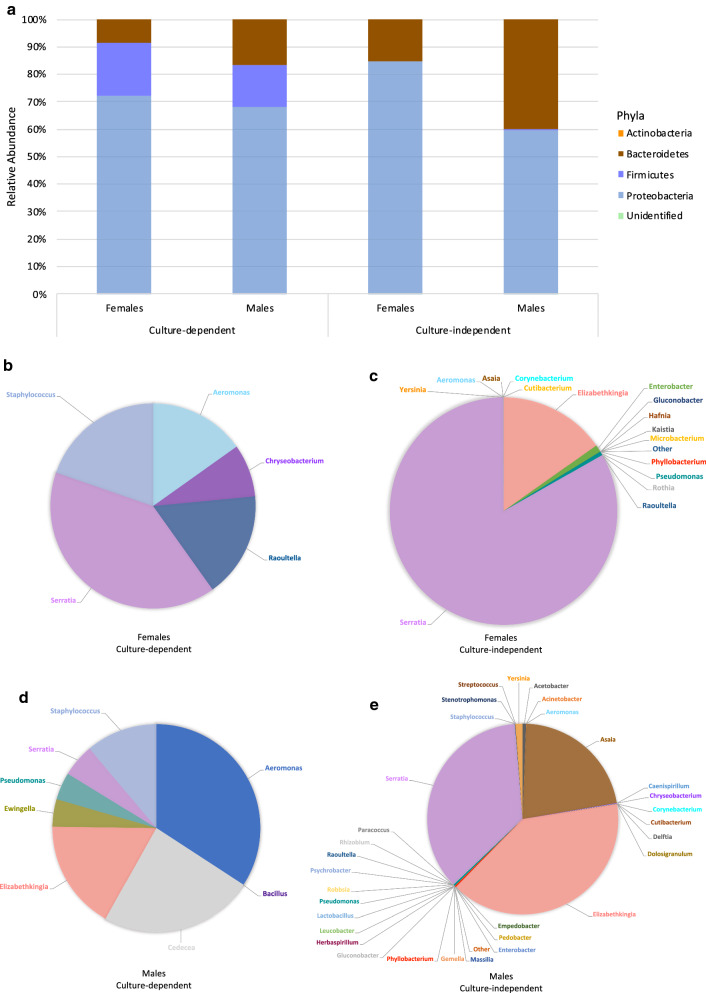
Relative abundance of *Anopheles funestus* microbial communities. Phyla are presented for **a** females and males, where each bar represents the average relative abundance identified either by culture-dependent or culture-independent techniques. Genera are presented for females identified by **b** culture-dependent and **c** culture-independent techniques and males identified by **d** culture-dependent and **e** culture-independent techniques

*Anopheles funestus* were predominantly colonised by Proteobacteria, irrespective of technique, followed by Bacteroidetes (Fig. 5a). While both identification techniques agree that *Serratia* is the dominant genus in females, culture-dependent results represent *Aeromonas* as the dominant genus in males and culture-independent results represent *Elizabethkingia* as the dominant genus in males (Fig. 5b–e).

**Fig. 6:**
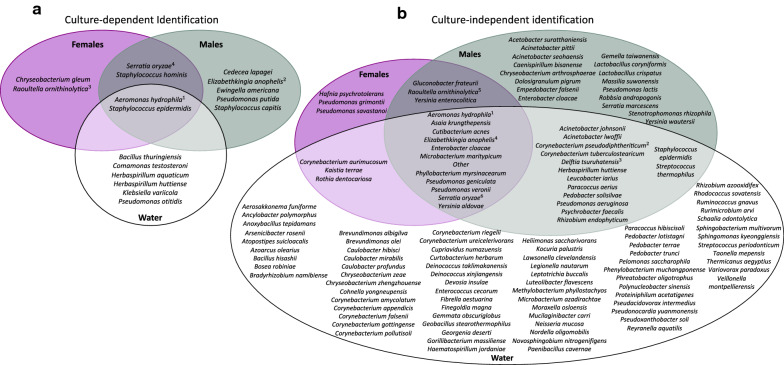
Bacteria identified by **a** culture-dependent and **b** culture-independent techniques from *Anopheles funestus* and the larval rearing water. Bacteria indistinguishable by MALDI-TOF MS include ^1^*A. hydrophila* and *A. veronii*; ^2^*C. propinquum* and *C. pseudodiphtheriticum*; ^3^*D. acidovorans* and *D. tsuruhatensis*; ^4^*E. anophelis*, *E. meningoseptica*, and *E. miricola*; ^5^* K. oxytoca*, *R. ornithinolytica*, *R. planticola*, and *R. terrigena*; and ^6^*S. fonticola* and *S. oryzae*

*Aeromonas hydrophila* was identified as a common bacterium between *An. funestus* females and males and their larval rearing water, irrespective of identification technique (Fig. 6). *Serratia oryzae* was common between both sexes in culture-dependent results but common in both sexes and the larval rearing water in culture-independent results. *Staphylococcus epidermidis* was common between both sexes and the larval rearing water in culture-dependent results but only in males and the larval rearing water in culture-independent results. Some bacteria were identified in a sex- and/or technique-dependent manner (see Additional file 8 for an overview of replicates).

Proteobacteria remained the dominant phylum in preserved *An. funestus* (Additional file 5). *Serratia oryzae* was a recurring bacterium that was common in silica-preserved and RNA*later*^®^-preserved *An. funestus* (Table 4; see Additional file 9 for replicate details). Irrespective of condition, *S. oryzae* was a recurring bacterium common between females and males. *Enterobacter cloacae* was specific to females and *S. epidermidis* was specific to males (Table 4; see Additional file 10 for replicate details). These sex-specific bacteria were also recurring bacteria in RNA*later*^®^-preserved (and not silica-preserved) samples. Overall, *S. oryzae* was a common bacterium between fresh and preserved *An. funestus*.

**Table 4:** Recurring bacteria identified in *Anopheles funestus* according to preservatives and sex

Bacteria	Silica-preserved	RNA*later*^®^-preserved	Females	Males
*Elizabethkingia anophelis*^*1*^	x			
*Enterobacter cloacae*		x	x	
*Phyllobacterium myrsinacearum*	x			
*Serratia oryzae*^*2*^	x	x	x	x
*Staphylococcus epidermidis*		x		x

Recurring bacteria appeared in at least half of all replicates per group. Bacteria indistinguishable by MALDI-TOF MS include ^1^*E. anophelis*, *E. meningoseptica*, and *E. miricola*; and ^2^*S. fonticola* and *S. oryzae*

### Comparison of microbial composition between *Anopheles arabiensis* and *Anopheles funestus*

Collectively, *An. arabiensis* and *An. funestus* were predominantly colonized by Proteobacteria (Additional file 11), irrespective of sex, condition or identification technique (see Additional file 12 for replicate details). Both species were also predominantly colonized by bacteria belonging to the *Elizabethkingia* and *Serratia* genera. *Elizabethkingia anophelis* and *S. oryzae* were recurring bacteria common between both species irrespective of sex, and *E. cloacae* was dominant in *An. funestus*. *Enterobacter cloacae* was specific to females, while *S. epidermidis* was specific to males, irrespective of species.

### Microbial diversity per sex, species, condition, and technique

Males of both species had higher bacterial species richness than females irrespective of species, condition or identification technique (Table 5). Nonetheless, *An. arabiensis* and *An. funestus* had overall comparable species richness.

**Table 5:** Bacterial species richness from fresh and preserved *Anopheles arabiensis* and *Anopheles funestus*

OTUs	Fresh				Preserved			
	Culture-dependent		Culture-independent		Culture-dependent		Culture-independent	
	*An. arabiensis*	*An. funestus*	*An. arabiensis*	*An. funestus*	*An. arabiensis*	*An. funestus*	*An. arabiensis*	*An. funestus*
Females	8	6	26	19	7	10	201	202
Males	9	10	33	42	13	11	245	233
Total	17	16	59	61	20	21	446	435

Species richness was identified by culture-dependent and culture-independent methods and measured as the number of OTUs

There were no significant differences amongst diversity indices between female and male mosquitoes (Additional file 13, 6A–C). There were also no significant differences amongst H and E indices between *An. arabiensis* and *An. funestus.* There was a significant difference in 1/D between species (P = 0.0209), where *An. arabiensis* had a higher 1/D than *An. funestus* (Additional file 13, 6D–F).

**Fig. 7:**
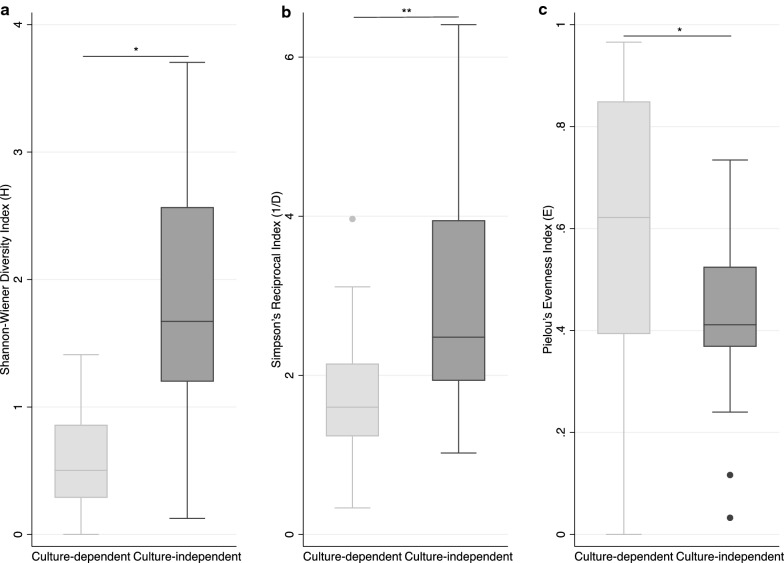
Box plots of diversity indices calculated for culture-dependent and culture-independent results. Upper and lower limits of boxes represent quartiles around the mean and horizontal lines within boxes represent median values for **a** Shannon–Wiener (H), **b** Simpson’s reciprocal (1/D), and **c** Pielou’s evenness (E). Significant differences were calculated with Wilcoxon rank-sum (Mann–Whitney) tests (*P < 0.05; **P < 0.01)

Diversity indices were comparable between fresh mosquitoes and mosquitoes preserved for 4 weeks (Additional file 14, 7A–C), fresh mosquitoes and mosquitoes preserved for 12 weeks (Additional file 14, 7D–F), and mosquitoes preserved for 4 and 12 weeks (Additional file 14, 7G–I). There were no significant differences amongst diversity indices between preservatives (Additional file 14, 7J–L). There were, however, significant differences in diversity indices between identification techniques. Culture-independent techniques estimated higher H (P = 0.0200) and 1/D (P = 0.0053) indices than culture-dependent techniques (Fig. 7A-B). Culture-dependent techniques estimated a higher E index (P = 0.0053) than culture-independent techniques (Fig. 7C).

## Discussion

Using culture-dependent and culture-independent techniques, this study characterized the microbiota of colonized adult *Anopheles* preserved either by desiccation in silica or fixation in RNA*later*^®^. This study echoes notions in the existing literature on the *Anopheles* microbiota: (i) culture-dependent and culture-independent techniques are complementary; (ii) microbiota are influenced by the mosquito’s environment (laboratory *vs* field); and, (iii) some microbiota are species- and/or sex-specific.

Colonized *Anopheles* in this study had abdomens predominated by Proteobacteria, which is the most common phylum reported in *Anopheles* studies [2, 5, 10, 18, 49, 53, 63, 122–124]. Previous studies have reported *Acinetobacter*, *Bacillus*, *Corynebacterium*, *Prevotella*, *Pseudomonas*, *Thorsellia*, and *Veillonella* as common genera in colonized *Anopheles*, while this study reported *Elizabethkingia* and *Serratia* as common genera in colonized *An. arabiensis* and *An. funestus* [8, 18, 39, 49, 88, 125]. Field-collected *An. arabiensis* were mainly colonized by Firmicutes, which corresponds with studies on the midguts of field-collected *Anopheles albimanus* from Colombia and *Anopheles* from Vietnam [53, 85]. These studies also identified *Staphylococcus* and *Bacillus* as dominant genera colonizing the midguts of field-caught *Anopheles*, which is consistent with the results of field-collected *An. arabiensis* in this study.

The dominant bacteria common between colonized *An. arabiensis* and *An. funestus* were *E. anophelis* and *S. oryzae. Elizabethkingia anophelis* was first isolated from *An. gambiae* midguts and has been well documented in *Anopheles* [126–128]. *Elizabethkingia anophelis* protects mosquitoes from infection and positively impacts fecundity in *Anopheles* [129, 130]. As *E. anophelis* was isolated from *An. arabiensis* and *An. funestus* in this study, this bacterium could have similar roles in both vector species. Meanwhile, *S. oryzae* has not been documented in *Anopheles* midguts and, thus, its role remains unknown.

*Staphylococcus epidermidis* was dominant in male *Anopheles* and has previously been isolated from the midguts of field-collected *Anopheles pharoensis* and from the salivary glands of colonized *An. arabiensis* [131]. However, its role is yet to be elucidated. *Enterobacter cloacae* was dominant in female *Anopheles*, as well as *An. funestus*, and is a known *Anopheles* midgut symbiont [132]. *Enterobacter cloacae* has been shown to influence vector immunity: *E. cloacae* affects the development of *Plasmodium berghei* and *P. falciparum* in *Anopheles stephensi*, as well as *Plasmodium vivax* in *An. albimanus* [48, 133]. Additionally, this bacterium has been tested for paratransgenesis, the genetic modification of symbiotic bacteria to express anti-*Plasmodium* effector molecules [134, 135], in *An. stephensi* [122]. Thus, *E. cloacae* may play an immunological role in female *Anopheles* and in *An. funestus*. The aforementioned bacteria could also have sex-specific roles in these anopheline species, possibly for the digestion of different food sources (i.e., digestion of blood by females and sugar by males) [16–18].

Furthermore, this study shows that neither condition (fresh or preserved), preservative (silica or RNA*later*^®^), nor preservation time-period (4 weeks or 12 weeks) influenced microbial composition or diversity. Thus, silica or RNA*later*^®^ are efficient, cost-effective alternatives to previously investigated preservatives; that is, AllProtect reagent, ethanol, and NAP buffer [93].

It was hypothesized that silica desiccation compacts the midgut to form a rigid, secure biofilm around the bacteria; previous studies report that bacterial species encapsulated in silica gel are preserved, along with their biological activity [136, 137]. During preservation, the bacteria enter a state of dormancy and when released into saline solution and plated on selective media, the bacteria exit dormancy and acquire nutrients for active growth. Therefore, using silica preservation in combination with culture-dependent techniques is useful because it distinguishes bacteria capable of entering and exiting dormancy, whereas culture-independent techniques cannot distinguish between live and dead bacteria [138]. However, silica preservation may only be suitable for specific bacteria.

It was also hypothesized that preserving mosquitoes in RNA*later*^®^ causes midguts to become pulpous, and RNA*later*^®^ solution may come into contact with the bacteria and inhibit growth when homogenates are placed on nutrient agar because RNA*later*^®^ is bacteriostatic [138, 139]. This may account for the few bacteria identified from RNA*later*^®^-preserved mosquitoes by culture-dependent techniques in comparison to culture-independent techniques. However, since RNA*later*^®^ preserves DNA in high quality, this preservation method is suitable when paired with culture-independent techniques.

Culture-independent techniques identified a richer and more diverse composition of bacteria than culture-dependent techniques, which is expected since culture-independent methods are highly sensitive [138]. As demonstrated, the type of bacterial identification technique affects microbial composition and diversity: culture-independent techniques estimated higher species richness, diversity, relative abundance, and microbial community evenness than culture-dependent technique. Yet, contamination cannot be ruled out.

Nevertheless, the combination of preservatives and identification methods is useful for identifying *Anopheles* midgut bacteria as it provides a large dataset of overlapping bacteria and can be used for future studies investigating fresh and preserved *Anopheles*. This may broaden the knowledge on the *Anopheles* microbial community and could aid future investigations elucidating the role that specific midgut bacteria play in vector species. It could also be used to compare the microbiota of preserved *P. falciparum*-infected and -uninfected vector species, thereby providing insight into the vector-microbiota-pathogen relationship.

A limit of this study is that the sample size of colonized mosquitoes was larger than the sample size of field-collected mosquitoes as the latter was scarce. Additionally, as there is no way of telling mosquito age upon collection, the age of field-collected *Anopheles* was unclear. Therefore, since microbiota change during development, the age of field-collected mosquitoes used in this study most likely influenced the bacteria identified [34, 140, 141]. The conditions that mosquitoes were handled during collection may have also affected the types of bacteria that were preserved (i.e., depending on the time it took for mosquitoes to be immobilised and placed on silica after field collection, this may have affected the bacterial community).

Although culture-independent procedures (DNA extraction and NGS) were performed at the same time for all samples, different generations of samples were collected. This may account for the difference in microbial composition between fresh and preserved samples. As culture-dependent techniques were performed at different times, but with the same generations of mosquitoes, this may have introduced batch effects. Thus, either generational effects, batch effects, contamination, or a combination of these, may account for the lack of uniformity amongst replicates.

Further, pooling does not provide a true representation of the mosquito microbiota because there is high variability between individual mosquitoes [93]. This may have also limited this study. The aforementioned limitations also limit the conclusions, and further investigation (investigating mosquito microbiota individually and increasing the overall sample sizes) is recommended.

As many midgut bacteria are acquired from the environment, identifying preserved microbiota from Diptera in an area can be used to study ecological changes in an environment over time (i.e., if there is a change in an environment, it would be worthwhile investigating if there is also a change in the microbiota of the Diptera inhabiting that area). This could aid in understanding changes in environmental bacteria and the effect that these changes have on the midguts of local Diptera, and on an ecological system as a whole. In addition, extending preservation studies using silica and RNA*later*^®^ to other Diptera may provide insight into Diptera-pathogen relationships and aid studies investigating symbiotic control to reduce disease transmission.

## Conclusions

This study shows that preserving *Anopheles* on silica or in RNA*later*^®^ for up to 12 weeks also preserves their microbiota. The findings of this study also demonstrate that silica- and RNA*later*^®^-preservation are appropriate when paired with culture-dependent and culture-independent techniques, respectively. These results broaden the selection of cost-effective preservatives for handling vector samples for downstream microbial analyses, especially as mosquito microbial studies begin to focus more on field-collected samples. This study also broadly characterized the *An. arabiensis* and *An. funestus* microbiota: *E. anophelis* and *S. oryzae* were dominant bacteria in both species, while *E. cloacae* and *S. epidermidis* were sex-specific bacteria. Future studies could investigate the role these bacteria play in anophelines, which could aid studies using the *Anopheles* microbiota to reduce malaria transmission in Africa.

## Supplementary Information

**Additional file 1. **OTUs with taxonomic assignment identified by culture-independent techniques. Samples have coded names: females are labelled ‘F.’, and males are labelled ‘M.’; larval rearing water is labelled ‘H2O’; *An. arabiensis* are labelled ‘Arab’, and *An. funestus* are labelled ‘Fun’; silica-preserved samples are labelled ‘S’, and RNA*later*^®^-preserved samples are labelled ‘R’; and samples preserved for 4 weeks begin with ‘1’, while samples preserved for 12 weeks begin with ‘3’. Each code ends with a number, either 1, 2 or 3, which denotes replicate numbers per group.

**Additional file 2. **Bacteria identified in this study. Bacteria were identified from fresh females and males and their larval rearing water, as well as from mosquitoes preserved on silica and in RNA*later*^®^ for up to 12 weeks, using culture-dependent (*) and culture-independent bacterial identification. Bacteria are recorded for all replicates (R). Bacteria indistinguishable by MALDI-TOF MS include ^1^*A. hydrophila* and *A. veronii*; ^2^*C. propinquum* and *C. pseudodiphtheriticum*; ^3^*D.* *acidovorans* and *D. tsuruhatensis*; ^4^*E. anophelis*, *E. meningoseptica*, and *E. miricola*; ^5^*E. coli* and *E. fergusonii*; ^6^*K. oxytoca*, *R. ornithinolytica*, *R. planticola*, and *R. terrigena*; and ^7^*S. fonticola* and *S. oryzae*.

**Additional file 3. **Diversity indices calculated in this study. Diversity indices were calculated for all replicates and average values were calculated per sex, species, preservative, preservation time period, condition (fresh *vs* preserved), and technique. The following indices were calculated: (A) Shannon-Wiener (H), (B) Simpson’s reciprocal (1/D), and (C) Pielou’s evenness (E).

**Additional file 4. **Bacteria identified by (A, B, C) culture-dependent and (D, E, F) culture-independent techniques from *Anopheles Arabiensis*. Bacteria were identified from fresh (A, D) females, (B, E) males, and (C, F) the larval rearing water. Bacteria indistinguishable by MALDI-TOF MS include ^1^*A. hydrophila* and *A. veronii*; ^2^*D. acidovorans* and *D. tsuruhatensis*; ^3^*E. anophelis*, *E. meningoseptica*, and *E. miricola*; ^4^*E. coli* and *E. fergusonii*; ^5^*K. oxytoca*, *R. ornithinolytica*, *R. planticola*, and *R. terrigena*; and ^6^*S. fonticola* and *S. oryzae*.

**Additional file 5. **Bacterial phyla identified by culture-dependent and culture-independent techniques from preserved (A) *Anopheles arabiensis* and (B) *Anopheles funestus*. Phyla are characterized according to sex (female or male), preservative (silica or in RNA*later*^®^), and preservation time period (4 weeks or 12 weeks).

**Additional file 6. **Bacteria identified from *Anopheles arabiensis* preserved on silica or in RNA*later*^®^. Bacteria were identified from females and males, as well as from mosquitoes preserved for 4 and 12 weeks, using culture-dependent (*) and culture-independent bacterial identification. Bacteria are recorded for all replicates (R). Bacteria indistinguishable by MALDI-TOF MS include ^1^*A. hydrophila* and *A. veronii*; ^2^*C. propinquum* and *C. pseudodiphtheriticum*; ^3^*D.* *acidovorans* and *D. tsuruhatensis*; ^4^*E. anophelis*, *E. meningoseptica*, and *E. miricola*; ^5^*E. coli* and *E. fergusonii*; ^6^*K. oxytoca*, *R. ornithinolytica*, *R. planticola*, and *R. terrigena*; and ^7^*S. fonticola* and *S. oryzae*.

**Additional file 7. **Bacteria identified from female and male *Anopheles arabiensis*. Bacteria were identified from fresh mosquitoes, as well as mosquitoes preserved for 4 and 12 weeks, using culture-dependent (*) and culture-independent bacterial identification. Bacteria are recorded for all replicates (R). Bacteria indistinguishable by MALDI-TOF MS include ^1^*A. hydrophila* and *A. veronii*; ^2^*C. propinquum* and *C. pseudodiphtheriticum*; ^3^*D.* *acidovorans* and *D. tsuruhatensis*; ^4^*E. anophelis*, *E. meningoseptica*, and *E. miricola*; ^5^*E. coli* and *E. fergusonii*; ^6^*K. oxytoca*, *R. ornithinolytica*, *R. planticola*, and *R. terrigena*; and ^7^*S. fonticola* and *S. oryzae*.

**Additional file 8. **Bacteria identified by (A, B, C) culture-dependent and (D, E, F) culture-independent techniques from *Anopheles funestus*. Bacteria were identified from fresh (A, D) females, (B, E) males, and (C, F) the larval rearing water. Bacteria indistinguishable by MALDI-TOF MS include ^1^*A. hydrophila *and *A. veronii*; ^2^*C. propinquum* and *C. pseudodiphtheriticum*; ^3^*D.* *acidovorans* and *D. tsuruhatensis*; ^4^*E. anophelis*, *E. meningoseptica*, and *E. miricola*; ^5^*K. oxytoca*, *R. ornithinolytica*, *R. planticola*, and *R. terrigena*; and ^6^*S. fonticola* and *S. oryzae*.

**Additional file 9. **Bacteria identified from *Anopheles funestus* preserved on silica or in RNA*later*^®^. Bacteria were identified from females and males, as well as from mosquitoes preserved for 4 and 12 weeks, using culture-dependent (*) and culture-independent bacterial identification. Bacteria are recorded for all replicates (R). Bacteria indistinguishable by MALDI-TOF MS include ^1^*A. hydrophila* and *A. veronii*; ^2^*C. propinquum* and *C. pseudodiphtheriticum*; ^3^*D.* *acidovorans* and *D. tsuruhatensis*; ^4^*E. anophelis*, *E. meningoseptica*, and *E. miricola*; ^5^*E. coli* and *E. fergusonii*, ^6^*K. oxytoca*, *R. ornithinolytica*, *R. planticola*, and *R. terrigena*; and ^7^*S. fonticola* and *S. oryzae*.

**Additional file 10. **Bacteria identified from female and male *Anopheles funestus*. Bacteria were identified from fresh mosquitoes, as well as mosquitoes preserved for 4 and 12 weeks, using culture-dependent (*) and culture-independent bacterial identification. Bacteria are recorded for all replicates (R). Bacteria indistinguishable by MALDI-TOF MS include ^1^*A. hydrophila *and* A. veronii*; ^2^*C. propinquum *and* C. pseudodiphtheriticum*; ^3^*D.* *acidovorans* and *D. tsuruhatensis*; ^4^*E. anophelis*, *E. meningoseptica*, and *E. miricola*; ^5^*E. coli* and *E. fergusonii*; ^6^*K. oxytoca*, *R. ornithinolytica*, *R. planticola*, and *R. terrigena*; and ^7^*S. fonticola* and *S. oryzae*.

**Additional file 11. **Accumulative bacterial phyla identified by culture-dependent and culture-independent techniques from preserved mosquitoes. Phyla are characterized according to species (*An. arabiensis* or *An. funestus*), sex (female or male), and preservative (silica or in RNA*later*^®^).

**Additional file 12. **Bacterial phyla identified from mosquitoes preserved for (A) 4 weeks and (B) 12 weeks. Phyla are characterized according to technique (culture-dependent or culture-independent), species (*An. arabiensis* or *An. funestus*), sex (female or male), and preservative (silica or in RNA*later*^®^). Phyla are recorded for all replicates (R).

**Additional file 13. **Box plots of diversity indices calculated for (A, B, C) sex and (D, E, F) species. Upper and lower limits of boxes represent quartiles around the mean and horizontal lines within boxes represent median values for (A, D) Shannon-Wiener (H), (B, E) Simpson’s reciprocal (1/D), and (C, F) Pielou’s evenness (E). Significant differences were calculated with Wilcoxon rank-sum (Mann-Whitney) tests (*P<0.05).

**Additional file 14. **Box plots of diversity indices comparing (A, B, C) fresh mosquitoes and mosquitoes preserved for 4 weeks (D, E, F) fresh mosquitoes and mosquitoes preserved for 12 weeks, (G, H, I) mosquitoes preserved for 4 weeks and mosquitoes preserved for 12 weeks, (J, K, L) silica- and RNA*later*^®^-preserved mosquitoes. Upper and lower limits of boxes represent quartiles around the mean and horizontal lines within boxes represent median values for (A, D, G, J) Shannon-Wiener (H), (B, E, H, K) Simpson’s reciprocal (1/D), and (C, F, I, L) Pielou’s evenness (E). Significant differences were calculated with Wilcoxon rank-sum (Mann-Whitney) tests.

## Data Availability

The datasets used and/or analysed are available from the corresponding author on reasonable request.

## Abbreviations

BHI: Brain–heart Infusion
BLASTn: Basic Local Alignment Search Tool for nucleotide sequences
1/D: Simpson’s Reciprocal Index
E: Pielou’s Evenness Index
EDTA: Ethylenediaminetetraacetic Acid
ELISA: Enzyme-linked Immunosorbent Assay
FLASH: Fast Length Adjustment of SHort Reads
H: Shannon–Wiener Diversity Index
HCCA: α-Cyano-4-hydroxycinnamic Acid
MALDI-TOF MS: Matrix-assisted Laser Desorption Ionization-Time of Flight Mass Spectrometry
NAP: Nucleic Acid Preservation
NGS: Next-generation Sequencing
NCBI: National Center for Biotechnology Information
NICD: National Institute for Communicable Diseases
OTU: Operational Taxonomic Unit
PBS: Phosphate-buffered Saline
PCR: Polymerase Chain Reaction
PMF: Peptide Mass Fingerprint
QIIME: Quantitative Insights Into Microbial Ecology
rRNA: Ribosomal Ribonucleic Acid
SIT: Sterile Insect Technique
WHO: World Health Organization

## References

[1] WHO. World Malaria Report 2020. Geneva: World Health Organization; 2020. https://www.who.int/teams/global-malaria-programme/reports/world-malaria-report-2020. Accessed Jan 2021.

[2] Boissière A, Tchioffo MT, Bachar D, Abate L, Marie A, Nsango SE (2012). Midgut microbiota of the malaria mosquito vector *Anopheles gambiae* and interactions with *Plasmodium falciparum* infection. PLoS Pathog..

[3] Chavshin AR, Oshaghi MA, Vatandoost H, Pourmand MR, Raeisi A, Terenius O (2014). Isolation and identification of culturable bacteria from wild *Anopheles culicifacies*, a first step in a paratransgenesis approach. Parasit Vectors.

[4] Coon KL, Vogel KJ, Brown MR, Strand MR (2014). Mosquitoes rely on their gut microbiota for development. Mol Ecol.

[5] Dennison NJ, Jupatanakul N, Dimopoulos G (2014). The mosquito microbiota influences vector competence for human pathogens. Curr Opin Insect Sci.

[6] Dickson LB, Ghozlane A, Volant S, Bouchier C, Ma L, Vega-Rúa A (2018). Diverse laboratory colonies of *Aedes aegypti* harbor the same adult midgut bacterial microbiome. Parasit Vectors.

[7] Dong Y, Manfredini F, Dimopoulos G (2009). Implication of the mosquito midgut microbiota in the defense against malaria parasites. PLoS Pathog..

[8] Lindh JM, Terenius O, Faye I (2005). 16S rRNA gene-based identification of midgut bacteria from field-caught *Anopheles gambiae sensu* lato and *A. funestus* mosquitoes reveals new species related to known insect symbionts. Appl Environ Microbiol..

[9] Merritt RW, Dadd RH, Walker ED (1992). Feeding behavior, natural food, and nutritional relationships of larval mosquitoes. Annu Rev Entomol.

[10] Osei-Poku J, Mbogo CM, Palmer WJ, Jiggins FM (2012). Deep sequencing reveals extensive variation in the gut microbiota of wild mosquitoes from Kenya. Mol Ecol.

[11] Saab SA, zu Dohna H, Nilsson LK, Onorati P, Nakhleh J, Terenius O (2020). The environment and species affect gut bacteria composition in laboratory co-cultured *Anopheles gambiae* and *Aedes albopictus* mosquitoes. Sci Rep..

[12] Steyn A, Roets F, Botha A (2016). Yeasts associated with *Culex pipiens* and *Culex theileri* mosquito larvae and the effect of selected yeast strains on the ontogeny of *Culex pipiens*. Microb Ecol.

[13] Wang S, Dos-Santos AL, Huang W, Liu KC, Oshaghi MA, Wei G (2017). Driving mosquito refractoriness to *Plasmodium falciparum* with engineered symbiotic bacteria. Science.

[14] Minard G, Mavingui P, Moro CV (2013). Diversity and function of bacterial microbiota in the mosquito holobiont. Parasit Vectors.

[15] Crotti E, Rizzi A, Chouaia B, Ricci I, Favia G, Alma A (2010). Acetic acid bacteria, newly emerging symbionts of insects. Appl Environ Microbiol.

[16] de O Gaio A, Gusmão DS, Santos AV, Berbert-Molina MA, Pimenta PF, Lemos FJ (2011). Contribution of midgut bacteria to blood digestion and egg production in *Aedes aegypti* (Diptera: Culicidae)(L.). Parasit Vectors..

[17] Gusmão DS, Santos AV, Marini DC, Bacci M, Berbert-Molina MA, Lemos FJ (2010). Culture-dependent and culture-independent characterization of microorganisms associated with *Aedes aegypti* (Diptera: Culicidae) (L.) and dynamics of bacterial colonization in the midgut. Acta Trop..

[18] Rani A, Sharma A, Rajagopal R, Adak T, Bhatnagar RK (2009). Bacterial diversity analysis of larvae and adult midgut microflora using culture-dependent and culture-independent methods in lab-reared and field-collected *Anopheles stephensi*-an Asian malarial vector. BMC Microbiol.

[19] Lindh JM, Kännaste A, Knols BG, Faye I, Borg-Karlson AK (2008). Oviposition responses of *Anopheles gambiae* s.s. (Diptera: Culicidae) and identification of volatiles from bacteria-containing solutions. J Med Entomol..

[20] Gendrin M, Rodgers FH, Yerbanga RS, Ouédraogo JB, Basáñez MG, Cohuet A (2015). Antibiotics in ingested human blood affect the mosquito microbiota and capacity to transmit malaria. Nat Commun.

[21] Verhulst NO, Beijleveld H, Knols BG, Takken W, Schraa G, Bouwmeester HJ (2009). Cultured skin microbiota attracts malaria mosquitoes. Malar J.

[22] Verhulst NO, Mukabana WR, Takken W, Smallegange RC (2011). Human skin microbiota and their volatiles as odour baits for the malaria mosquito *Anopheles gambiae* s.s. Entomol Exp Appl..

[23] Barnard K, Jeanrenaud AC, Brooke BD, Oliver SV (2019). The contribution of gut bacteria to insecticide resistance and the life histories of the major malaria vector *Anopheles arabiensis* (Diptera: Culicidae). Sci Rep.

[24] Dada N, Sheth M, Liebman K, Pinto J, Lenhart A (2018). Whole metagenome sequencing reveals links between mosquito microbiota and insecticide resistance in malaria vectors. Sci Rep.

[25] Dada N, Lol JC, Benedict AC, López F, Sheth M, Dzuris N (2019). Pyrethroid exposure alters internal and cuticle surface bacterial communities in *Anopheles albimanus*. ISME J.

[26] Kwon GS, Sohn HY, Shin KS, Kim E, Seo BI (2005). Biodegradation of the organochlorine insecticide, endosulfan, and the toxic metabolite, endosulfan sulfate, by *Klebsiella oxytoca KE-8*. Appl Microbiol Biotechnol.

[27] Soltani A, Vatandoost H, Oshaghi MA, Enayati AA, Chavshin AR (2017). The role of midgut symbiotic bacteria in resistance of *Anopheles stephensi* (Diptera: Culicidae) to organophosphate insecticides. Pathog Glob Health.

[28] Chouaia B, Rossi P, Epis S, Mosca M, Ricci I, Damiani C (2012). Delayed larval development in *Anophele*s mosquitoes deprived of *Asaia* bacterial symbionts. BMC Microbiol.

[29] Jadin J, Vincke IH, Dunjic A, Delville JP, Wery M, Bafort J (1967). Role of *Pseudomonas* in the sporogenesis of the hematozoon of malaria in the mosquito. Bull Soc Pathol Exot.

[30] Mitraka E, Stathopoulos S, Siden-Kiamos I, Christophides GK, Louis C (2013). *Asaia* accelerates larval development of *Anopheles gambiae*. Pathog Glob Health.

[31] Pumpuni CB, Beier MS, Nataro JP, Guers LD, Davis JR (1993). *Plasmodium falciparum*: inhibition of sporogonic development in *Anopheles stephensi* by gram-negative bacteria. Exp Parasitol.

[32] Rodgers FH, Gendrin M, Wyer CA, Christophides GK (2017). Microbiota-induced peritrophic matrix regulates midgut homeostasis and prevents systemic infection of malaria vector mosquitoes. PLoS Pathog..

[33] Seitz HM, Maier WA, Rottok M, Becker-Feldmann H (1987). Concomitant infections of *Anopheles stephensi* with *Plasmodium berghei* and *Serratia marcescens*: additive detrimental effects. Zentralbl Bakteriol Mikrobiol Hyg A.

[34] Wang Y, Gilbreath TM 3rd, Kukutla P, Yan G, Xu J. Dynamic gut microbiome across life history of the malaria mosquito *Anopheles gambiae* in Kenya. PLoS One. 2011;6:e24767. 10.1371/journal.pone.0024767 PMC3177825 21957459

[35] Wotton RS, Chaloner DT, Yardley CA, Merritt RW (1997). Growth of *Anopheles* mosquito larvae on dietary microbiota in aquatic surface microlayers. Med Vet Entomol.

[36] Favia G, Ricci I, Damiani C, Raddadi N, Crotti E, Marzorati M (2007). Bacteria of the genus *Asaia* stably associate with *Anopheles stephensi*, an Asian malarial mosquito vector. Proc Natl Acad Sci USA.

[37] Bahia AC, Dong Y, Blumberg BJ, Mlambo G, Tripathi A, BenMarzouk-Hidalgo OJ (2014). Exploring *Anopheles* gut bacteria for *Plasmodium* blocking activity. Environ Microbiol.

[38] Blumberg BJ, Trop S, Das S, Dimopoulos G (2013). Bacteria- and IMD pathway-independent immune defenses against *Plasmodium falciparum* in *Anopheles gambiae*. PLoS ONE.

[39] Briones AM, Shililu J, Githure J, Novak R, Raskin L (2008). *Thorsellia anophelis* is the dominant bacterium in a Kenyan population of adult *Anopheles gambiae* mosquitoes. ISME J.

[40] Cirimotich CM, Dong Y, Clayton AM, Sandiford SL, Souza-Neto JA, Mulenga M (2011). Natural microbe-mediated refractoriness to *Plasmodium* infection in *Anopheles gambiae*. Science.

[41] Dennison NJ, BenMarzouk-Hidalgo OJ, Dimopoulos G (2015). MicroRNA-regulation of *Anopheles gambiae* immunity to *Plasmodium falciparum* infection and midgut microbiota. Dev Comp Immunol.

[42] Dennison NJ, Saraiva RG, Cirimotich CM, Mlambo G, Mongodin EF, Dimopoulos G (2016). Functional genomic analyses of *Enterobacter*, *Anopheles* and *Plasmodium* reciprocal interactions that impact vector competence. Malar J.

[43] Dong Y, Aguilar R, Xi Z, Warr E, Mongin E, Dimopoulos G (2006). *Anopheles gambiae* immune responses to human and rodent *Plasmodium* parasite species. PLoS Pathog..

[44] Favia G, Ricci I, Marzorati M, Negri I, Alma A, Sacchi L (2008). Bacteria of the genus *Asaia*: a potential paratransgenic weapon against malaria. Adv Exp Med Biol.

[45] Garver LS, Bahia AC, Das S, Souza-Neto JA, Shiao J, Dong Y (2012). *Anopheles* Imd pathway factors and effectors in infection intensity-dependent anti-*Plasmodium* action. PLoS Pathog..

[46] Gendrin M, Christophides GK. The *Anopheles* mosquito microbiota and their impact on pathogen transmission. In: *Anopheles* mosquitoes—new insights into malaria vectors, Manguin. S. Intech; 2013. https://www.intechopen.com/books/anopheles-mosquitoes-new-insights-into-malaria-vectors/the-anopheles-mosquito-microbiota-and-their-impact-on-pathogen-transmission. Accessed 12 May 2020.

[47] Gendrin M, Turlure F, Rodgers FH, Cohuet A, Morlais I, Christophides GK (2017). The peptidoglycan recognition proteins PGRPLA and PGRPLB regulate *Anopheles* immunity to bacteria and affect infection by *Plasmodium*. J Innate Immun.

[48] Gonzalez-Ceron L, Santillan F, Rodriguez MH, Mendez D, Hernandez-Avila JE (2003). Bacteria in midguts of field-collected *Anopheles albimanus* block *Plasmodium vivax* sporogonic development. J Med Entomol.

[49] Kalappa DM, Subramani PA, Basavanna SK, Ghosh SK, Sundaramurthy V, Uragayala S (2018). Influence of midgut microbiota in *Anopheles stephensi* on *Plasmodium berghei* infections. Malar J.

[50] Meister S, Agianian B, Turlure F, Relógio A, Morlais I, Kafatos FC (2009). *Anopheles gambiae* PGRPLC-mediated defense against bacteria modulates infections with malaria parasites. PLoS Pathog..

[51] Michel K, Budd A, Pinto S, Gibson TJ, Kafatos FC (2005). *Anopheles gambiae* SRPN2 facilitates midgut invasion by the malaria parasite *Plasmodium berghei*. EMBO Rep.

[52] Ngo CT, Aujoulat F, Veas F, Jumas-Bilak E, Manguin S (2015). Bacterial diversity associated with wild caught *Anopheles* mosquitoes from Dak Nong Province, Vietnam using culture and DNA fingerprint. PLoS ONE.

[53] Ngo CT, Romano-Bertrand S, Manguin S, Jumas-Bilak E (2016). Diversity of the bacterial microbiota of *Anopheles* mosquitoes from Binh Phuoc Province. Vietnam Front Microbiol.

[54] Ramirez JL, Short SM, Bahia AC, Saraiva RG, Dong Y, Kang S (2014). *Chromobacterium* Csp_P reduces malaria and dengue infection in vector mosquitoes and has entomopathogenic and in vitro anti-pathogen activities. PLoS Pathog..

[55] Rodrigues J, Brayner FA, Alves LC, Dixit R, Barillas-Mury C (2010). Hemocyte differentiation mediates innate immune memory in *Anopheles gambiae* mosquitoes. Science.

[56] Ross R (1911). The prevention of malaria.

[57] Saraiva RG, Kang S, Simoes ML, Angleró-Rodríguez YI, Dimopoulos G (2016). Mosquito gut antiparasitic and antiviral immunity. Dev Comp Immunol.

[58] Smith RC, Vega-Rodríguez J, Jacobs-Lorena M (2014). The *Plasmodium* bottleneck: malaria parasite losses in the mosquito vector. Mem Inst Oswaldo Cruz.

[59] Straif SC, Mbogo CN, Toure AM, Walker ED, Kaufman M, Toure YT (1998). Midgut bacteria in *Anopheles gambiae* and *An. funestus* (Diptera: Culicidae) from Kenya and Mali. J Med Entomol..

[60] Stathopoulos S, Neafsey DE, Lawniczak MK, Muskavitch MA, Christophides GK (2014). Genetic dissection of *Anopheles gambiae* gut epithelial responses to *Serratia marcescens*. PLoS Pathog..

[61] Tchioffo MT, Boissière A, Churcher TS, Abate L, Gimonneau G, Nsango SE (2013). Modulation of malaria infection in *Anopheles gambiae* mosquitoes exposed to natural midgut bacteria. PLoS ONE.

[62] Tchioffo MT, Boissière A, Abate L, Nsango SE, Bayibéki AN, Awono-Ambéné PH (2016). Dynamics of bacterial community composition in the malaria mosquito's epithelia. Front Microbiol.

[63] Volohonsky G, Hopp AK, Saenger M, Soichot J, Scholze H, Boch J (2017). Transgenic expression of the anti-parasitic factor TEP1 in the malaria mosquito *Anopheles gambiae*. PLoS Pathog..

[64] Wang S, Ghosh AK, Bongio N, Stebbings KA, Lampe DJ, Jacobs-Lorena M (2012). Fighting malaria with engineered symbiotic bacteria from vector mosquitoes. Proc Natl Acad Sci USA.

[65] Wang S, Jacobs-Lorena M (2013). Genetic approaches to interfere with malaria transmission by vector mosquitoes. Trends Biotechnol.

[66] Wang S, Bai L, Wang L, Vega-Rodríguez J, Wang G (2019). A gut symbiotic bacterium *Serratia marcescens* renders mosquito resistance to *Plasmodium infection* through activation of mosquito immune responses. Front Microbiol.

[67] Zhang G, Niu G, Franca CM, Dong Y, Wang X, Butler NS (2015). *Anopheles* midgut FREP1 mediates *Plasmodium* invasion. J Biol Chem.

[68] Tandina F, Almeras L, Koné AK, Doumbo OK, Raoult D, Parola P (2016). Use of MALDI-TOF MS and culturomics to identify mosquitoes and their midgut microbiota. Parasit Vectors.

[69] Fall B, Lo CI, Samb-Ba B, Perrot N, Diawara S, Gueye MW (2015). The ongoing revolution of MALDI-TOF mass spectrometry for microbiology reaches tropical Africa. Am J Trop Med Hyg.

[70] Karger A (2016). Current developments to use linear MALDI-TOF spectra for the identification and typing of bacteria and the characterization of other cells/organisms related to infectious diseases. Proteomics Clin Appl.

[71] Singhal N, Kumar M, Kanaujia PK, Virdi JS (2015). MALDI-TOF mass spectrometry: an emerging technology for microbial identification and diagnosis. Front Microbiol.

[72] Lagier JC, Armougom F, Million M, Hugon P, Pagnier I, Robert C (2012). Microbial culturomics: paradigm shift in the human gut microbiome study. Clin Microbiol Infect.

[73] Cobo F (2013). Application of MALDI-TOF mass spectrometry in clinical virology: a review. Open Virol J.

[74] Boers SA, Jansen R, Hays JP (2019). Understanding and overcoming the pitfalls and biases of next-generation sequencing (NGS) methods for use in the routine clinical microbiological diagnostic laboratory. Eur J Clin Microbiol Infect Dis.

[75] Kim M, Morrison M, Yu Z (2011). Evaluation of different partial 16S rRNA gene sequence regions for phylogenetic analysis of microbiomes. J Microbiol Methods.

[76] Tringe SG, Hugenholtz P (2008). A renaissance for the pioneering 16S rRNA gene. Curr Opin Microbiol.

[77] Wang Y, Qian PY (2009). Conservative fragments in bacterial 16S rRNA genes and primer design for 16S ribosomal DNA amplicons in metagenomic studies. PLoS ONE.

[78] Bizzini A, Jaton K, Romo D, Bille J, Prod'hom G, Greub G (2011). Matrix-assisted laser desorption ionization–time of flight mass spectrometry as an alternative to 16S rRNA gene sequencing for identification of difficult-to-identify bacterial strains. J Clin Microbiol.

[79] Cherkaoui A, Hibbs J, Emonet S, Tangomo M, Girard M, Francois P (2010). Comparison of two matrix-assisted laser desorption ionization-time of flight mass spectrometry methods with conventional phenotypic identification for routine identification of bacteria to the species level. J Clin Microbiol.

[80] Ross MG, Russ C, Costello M, Hollinger A, Lennon NJ, Hegarty R (2013). Characterizing and measuring bias in sequence data. Genome Biol.

[81] Seng P, Drancourt M, Gouriet F, La Scola B, Fournier PE, Rolain JM (2009). Ongoing revolution in bacteriology: routine identification of bacteria by matrix-assisted laser desorption ionization time-of-flight mass spectrometry. Clin Infect Dis.

[82] Pandya SKR, Srinivas V, Jadhav S, Khan A, Arun A, Riley LW (2017). Comparison of culture-dependent and culture-independent molecular methods for characterization of vaginal microflora. J Med Microbiol.

[83] Djadid ND, Jazayeri H, Raz A, Favia G, Ricci I, Zakeri S (2011). Identification of the midgut microbiota of *An. stephensi* and *An. maculipennis* for their application as a paratransgenic tool against malaria. PLoS ONE.

[84] Chavshin AR, Oshaghi MA, Vatandoost H, Pourmand MR, Raeisi A, Enayati AA (2012). Identification of bacterial microflora in the midgut of the larvae and adult of wild caught *Anopheles stephensi*: a step toward finding suitable paratransgenesis candidates. Acta Trop.

[85] Galeano-Castañeda Y, Urrea-Aguirre P, Piedrahita S, Bascuñán P, Correa MM (2019). Composition and structure of the culturable gut bacterial communities in *Anopheles albimanus* from Colombia. PLoS ONE.

[86] Magurran AE (1988). Ecological diversity and its measurement.

[87] Hill TC, Walsh KA, Harris JA, Moffett BF (2003). Using ecological diversity measures with bacterial communities. FEMS Microbiol Ecol.

[88] Akorli J, Gendrin M, Pels NA, Yeboah-Manu D, Christophides GK, Wilson MD (2016). Seasonality and locality affect the diversity of *Anopheles gambiae* and *Anopheles coluzzii* midgut microbiota from Ghana. PLoS ONE.

[89] Dowell FE, Noutcha AE, Michel K (2011). The effect of preservation methods on predicting mosquito age by near Infrared spectroscopy. Am J Trop Med Hyg.

[90] Camacho-Sanchez M, Burraco P, Gomez-Mestre I, Leonard JA (2013). Preservation of RNA and DNA from mammal samples under field conditions. Mol Ecol Resources.

[91] Hunt RH, Coetzee M (1986). Field sampling of *Anopheles* mosquitos for correlated cytogenic, electrophoretic and morphological studies. Bull World Health Organ.

[92] Mayagaya VS, Ntamatungiro AJ, Moore SJ, Wirtz RA, Dowell FE, Maia MF (2015). Evaluating preservation methods for identifying *Anopheles gambiae* s.s. and *Anopheles arabiensis* complex mosquitoes species using near infra-red spectroscopy. Parasit Vectors..

[93] Rodríguez-Ruano SM, Juhaňáková E, Vávra J, Nováková E (2020). Methodological insight into mosquito microbiome studies. Front Cell Infect Microbiol.

[94] Zawada JW, Dahan-Moss YL, Muleba M, Dabire RK, Maïga H, Venter N (2018). Molecular and physiological analysis of *Anopheles funestus* swarms in Nchelenge. Zambia Malar J.

[95] Quicke DL, Lopez-Vaamonde C, Belshaw R (1999). Preservation of hymenopteran specimens for subsequent molecular and morphological study. Zool Scr.

[96] Hargreaves K, Hunt RH, Brooke BD, Mthembu J, Weeto MM, Awolola TS (2003). *Anopheles arabiensis* and *An. quadriannulatus* resistance to DDT in South Africa. Med Vet Entomol..

[97] Hunt RH, Brooke BD, Pillay C, Koekemoer LL, Coetzee M (2005). Laboratory selection for and characteristics of pyrethroid resistance in the malaria vector *Anopheles funestus*. Med Vet Entomol.

[98] Scott JA, Brogdon WG, Collins FH (1993). Identification of single specimens of the *Anopheles gambiae* complex by the polymerase chain reaction. Am J Trop Med Hyg.

[99] Koekemoer LL, Kamau L, Hunt RH, Coetzee M (2002). A cocktail polymerase chain reaction assay to identify members of the *Anopheles funestus* (Diptera: Culicidae) group. Am J Trop Med Hyg.

[100] Cohuet A, Simard F, Toto JC, Kengne P, Coetzee M, Fontenille D (2003). Species identification within the *Anopheles funestus* group of malaria vectors in Cameroon and evidence for a new species. Am J Trop Med Hyg.

[101] Wirtz RA, Zavala F, Charoenvit Y, Campbell GH, Burkot TR, Schneider I (1987). Comparative testing of monoclonal antibodies against *Plasmodium falciparum* sporozoites for ELISA development. Bull World Health Organ.

[102] Dandalo LC, Brooke BD, Munhenga G, Lobb LN, Zikhali J, Ngxongo SP (2017). Population dynamics and *Plasmodium falciparum* (Haemosporida: Plasmodiidae) infectivity rates for the malaria vector *Anopheles arabiensis* (Diptera: Culicidae) at Mamfene, KwaZulu-Natal. South Africa J Med Entomol.

[103] South African Weather Service. https://www.weathersa.co.za/home/historicalrain. Accessed 14 Oct 2020.

[104] WHO (1963). Practical entomology in malaria eradication.

[105] MacConkey A (1905). Lactose-fermenting bacteria in faeces. J Hyg (Lond).

[106] Murray PR, Baron EJ, Pfaller MA, Tenover FC, Yolken RH, Greenwood D (1996). Manual of clinical microbiology (6th edn). Trends Microbiol..

[107] Ferrieri P, Blair LL (1977). Pharyngeal carriage of group B streptococci: detection by three methods. J Clin Microbiol.

[108] Chapman GH, Lieb CW, Berens C, Curcio L (1937). The isolation of probable pathogenic staphylococci. J Bacteriol.

[109] Rosenow EC (1919). Studies on elective localization focal infection with special reference to oral sepsis. J Dent Res.

[110] Herlemann DP, Labrenz M, Jürgens K, Bertilsson S, Waniek JJ, Andersson AF (2011). Transitions in bacterial communities along the 2000 km salinity gradient of the Baltic Sea. ISME J.

[111] Magoč T, Salzberg SL (2011). FLASH: fast length adjustment of short reads to improve genome assemblies. Bioinformatics.

[112] Li W, Fu L, Niu B, Wu S, Wooley J (2012). Ultrafast clustering algorithms for metagenomic sequence analysis. Brief Bioinform.

[113] Schloss PD, Westcott SL, Ryabin T, Hall JR, Hartmann M, Hollister EB (2009). Introducing mothur: open-source, platform-independent, community-supported software for describing and comparing microbial communities. Appl Environ Microbiol.

[114] Caporaso JG, Kuczynski J, Stombaugh J, Bittinger K, Bushman FD, Costello EK (2010). QIIME allows analysis of high-throughput community sequencing data. Nat Methods.

[115] Lin JN, Lai CH, Yang CH, Huang YH (2018). Comparison of clinical manifestations, antimicrobial susceptibility patterns, and mutations of fluoroquinolone target genes between *Elizabethkingia meningoseptica* and *Elizabethkingia anophelis* isolated in Taiwan. J Clin Med.

[116] Sitaula S, Shahrrava A, Al Zoubi M, Malow J (2016). The first case report of *Raoultella planticola* liver abscess. IDCases.

[117] Surani A, Slama EM, Thomas S, Ross RW, Cunningham SC (2020). *Raoultella ornithinolytica* and *Klebsiella oxytoca* pyogenic liver abscess presenting as chronic cough. IDCases..

[118] Shannon CE (1948). A mathematical theory of communication. Bell Syst Tech J.

[119] Simpson EH (1949). Measurement of diversity. Nature.

[120] Pielou EC (1975). Ecological diversity.

[121] Zhang Z, Schwartz S, Wagner L, Miller W (2000). A greedy algorithm for aligning DNA sequences. J Comput Biol.

[122] Cirimotich CM, Ramirez JL, Dimopoulos G (2011). Native microbiota shape insect vector competence for human pathogens. Cell Host Microbe.

[123] Gonçalves GG, Feitosa AP, Portela-Júnior NC, de Oliveira CM, de Lima Filho JL, Brayner FA (2019). Use of MALDI-TOF MS to identify the culturable midgut microbiota of laboratory and wild mosquitoes. Acta Trop..

[124] Nilsson LK, de Oliveira MR, Marinotti O, Rocha EM, Håkansson S, Tadei WP (2019). Characterization of bacterial communities in breeding waters of *Anopheles darlingi* in Manaus in the Amazon Basin malaria-endemic area. Microb Ecol.

[125] Villegas LM, Pimenta PF (2014). Metagenomics, paratransgenesis and the *Anopheles* microbiome: a portrait of the geographical distribution of the anopheline microbiota based on a meta-analysis of reported taxa. Mem Inst Oswaldo Cruz.

[126] Chen S, Bagdasarian M, Walker ED (2015). *Elizabethkingia anophelis*: molecular manipulation and interactions with mosquito hosts. Appl Environ Microbiol.

[127] Kämpfer P, Matthews H, Glaeser SP, Martin K, Lodders N, Faye I. *Elizabethkingia anophelis* sp. nov., isolated from the midgut of the mosquito *Anopheles gambiae.* Int J Syst Evol Microbiol. 2011;61:2670–5. 10.1099/ijs.0.026393-0 21169462

[128] Kukutla P, Lindberg BG, Pei D, Rayl M, Yu W, Steritz M (2014). Insights from the genome annotation of *Elizabethkingia anophelis* from the malaria vector *Anopheles gambiae*. PLoS ONE.

[129] Akhouayri IG, Habtewold T, Christophides GK (2013). Melanotic pathology and vertical transmission of the gut commensal *Elizabethkingia meningoseptica* in the major malaria vector *Anopheles gambiae*. PLoS ONE.

[130] Chen S, Johnson BK, Yu T, Nelson BN, Walker ED (2020). *Elizabethkingia anophelis*: physiologic and transcriptomic responses to iron stress. Front Microbiol.

[131] Terenius O, De Oliveira CD, Pinheiro WD, Tadei WP, James AA, Marinotti O (2008). 16S rRNA gene sequences from bacteria associated with adult *Anopheles darlingi* (Diptera: Culicidae) mosquitoes. J Med Entomol.

[132] Berhanu A, Abera A, Nega D, Mekasha S, Fentaw S, Assefa A (2019). Isolation and identification of microflora from the midgut and salivary glands of *Anopheles* species in malaria endemic areas of Ethiopia. BMC Microbiol.

[133] Eappen A, Smith R, Jacobs-Lorena M (2013). *Enterobacter*-activated mosquito immune responses to *Plasmodium* involve activation of SRPN6 in *Anopheles stephensi*. PLoS ONE.

[134] Wilke AB, Marrelli MT (2015). Paratransgenesis: a promising new strategy for mosquito vector control. Parasit Vectors.

[135] Dehghan H, Oshaghi MA, Moosa-Kazemi SH, Yakhchali B, Vatandoost H, Maleki-Ravasan N (2017). Dynamics of transgenic *Enterobacter cloacae* expressing green fluorescent protein defensin (GFP-D) in *Anopheles stephensi* under laboratory condition. J Arthropod Borne Dis.

[136] Grivell AR, Jackson JF (1969). Microbial culture preservation with silica gel. J Gen Microbiol.

[137] Mutlu BR, Hirschey K, Wackett LP, Aksan A (2015). Long-term preservation of silica gel-encapsulated bacterial biocatalysts by desiccation. J Sol-Gel Sci Technol.

[138] Figdor D, Gulabivala K (2008). Survival against the odds: microbiology of root canals associated with post-treatment disease. Endod Topics.

[139] Sigma-Aldrich Technical Bulletin, 2016. RNA Stabilization Solution for Tissue. https://www.sigmaaldrich.com/content/dam/sigma-aldrich/docs/Sigma/Bulletin/1/r0901bul.pdf. Accessed 4 Nov 2020.

[140] Gimonneau G, Tchioffo MT, Abate L, Boissière A, Awono-Ambéné PH, Nsango SE (2014). Composition of *Anopheles coluzzii* and *Anopheles gambiae* microbiota from larval to adult stages. Infect Genet Evol.

[141] Kim CH, Lampman RL, Muturi EJ (2015). Bacterial communities and midgut microbiota associated with mosquito populations from waste tires in East-Central Illinois. J Med Entomol.

