# Supplementary figures and images for: Microbiota identified from preserved Anopheles
Source: Malar J. 2021 May 22;20:230. doi: 10.1186/s12936-021-03754-7 (PMC8141131; doi:10.1186/s12936-021-03754-7)

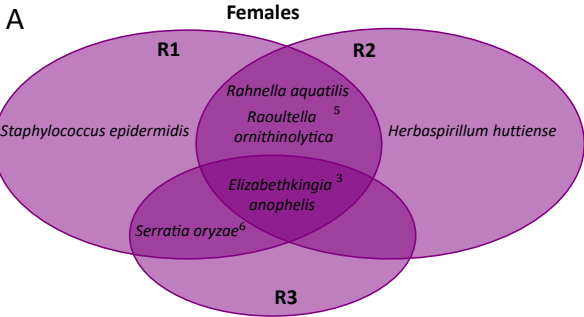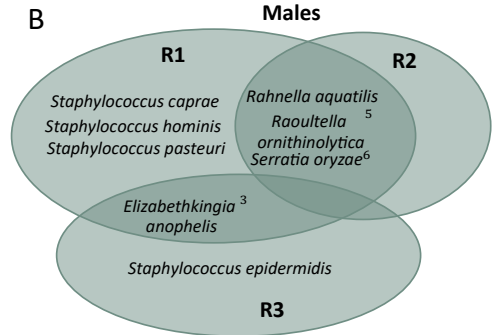

Culture-dependent Identification

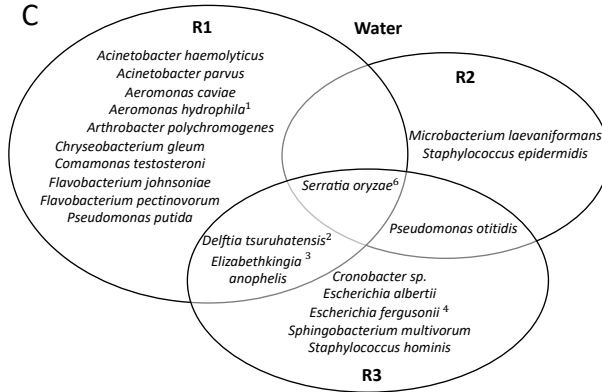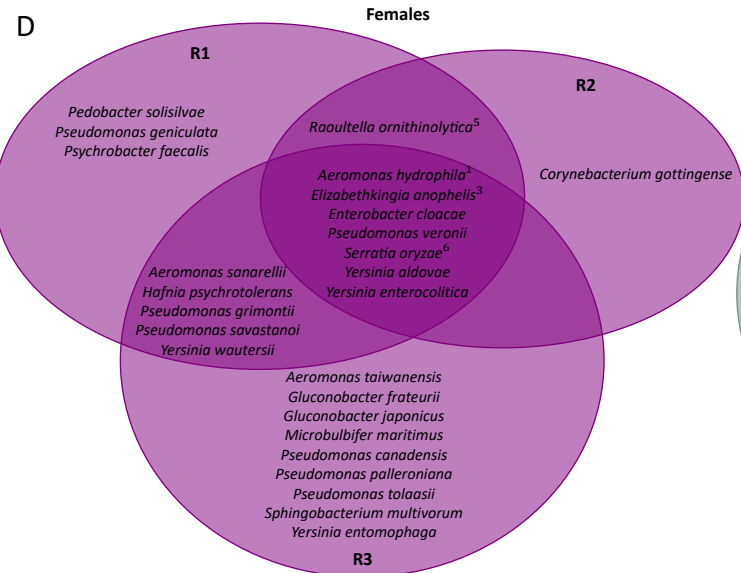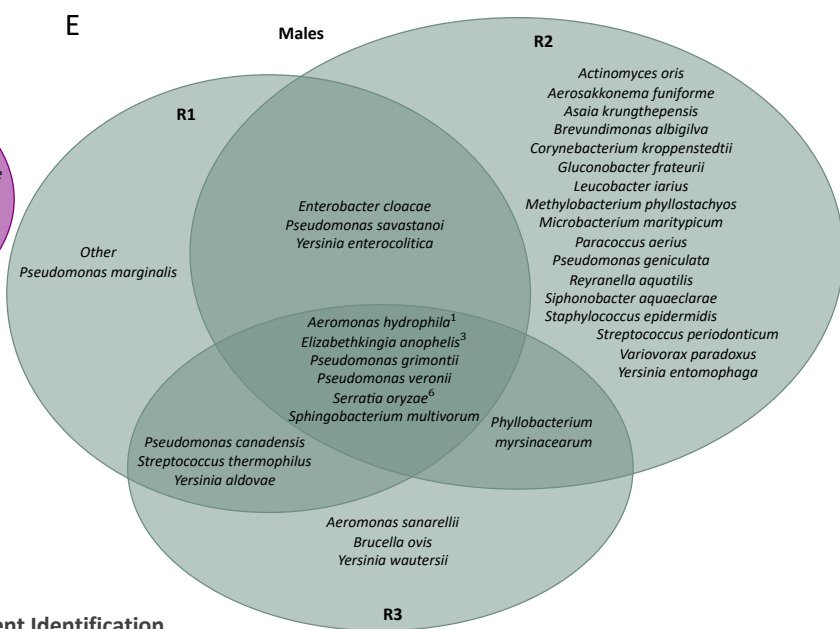

Culture-independent Identification

Water

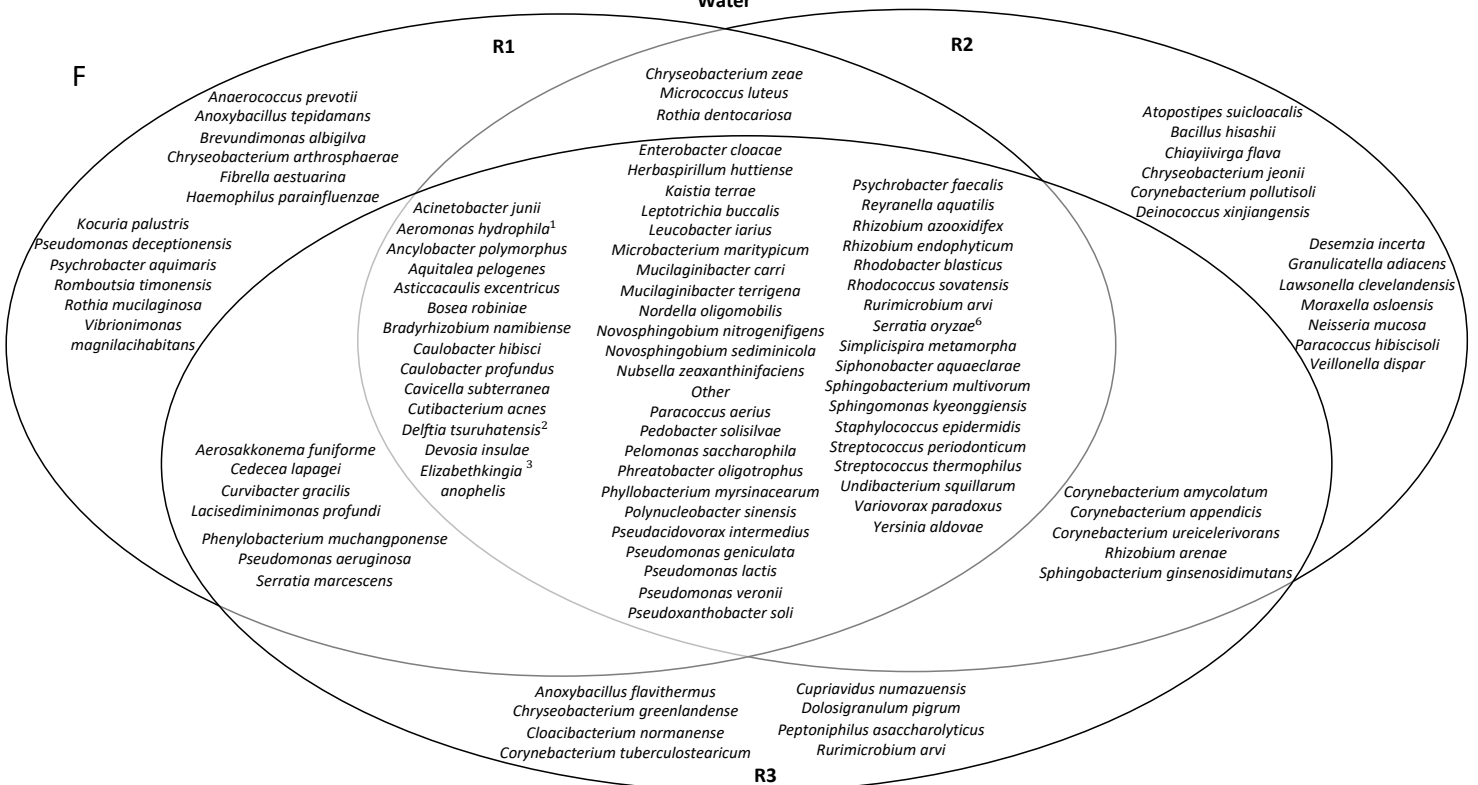

Supplement: Supplementary file 4 — Additional file 4. Bacteria identified by (A, B, C) culture-dependent and (D, E, F) culture-independent techniques from Anopheles Arabiensis. Bacteria were identified from fresh (A, D) females, (B, E) males, and (C, F) the larval rearing water. Bacteria indistinguishable by MALDI-TOF MS include 1A. hydrophila and A. veronii; 2D. acidovorans and D. tsuruhatensis; 3E. anophelis, E. meningoseptica, and E. miricola; 4E. coli and E. fergusonii; 5K. oxytoca, R. ornithinolytica, R. planticola, and R. terrigena; and 6S. fonticola and S. oryzae. [file 12936_2021_3754_MOESM4_ESM.pdf]

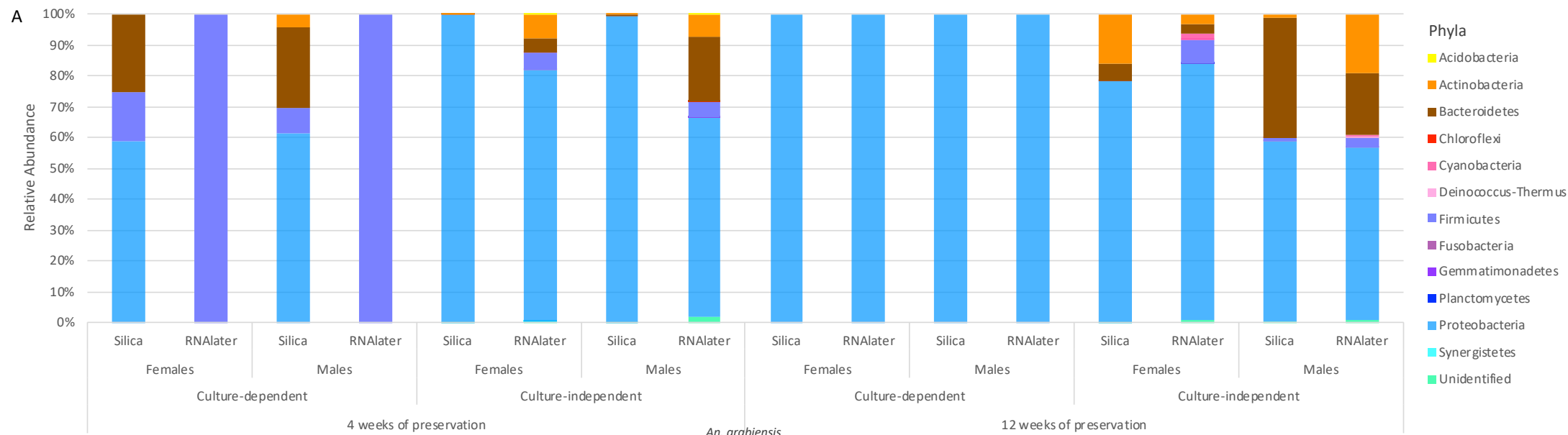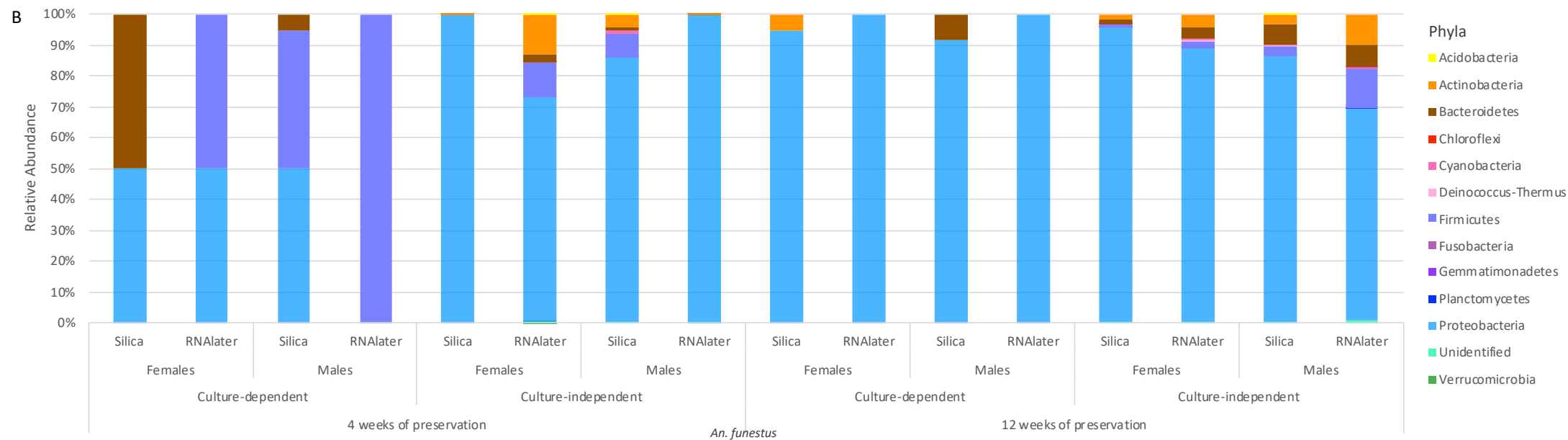

Supplement: Supplementary file 5 — Additional file 5. Bacterial phyla identified by culture-dependent and culture-independent techniques from preserved (A) Anopheles arabiensis and (B) Anopheles funestus. Phyla are characterized according to sex (female or male), preservative (silica or in RNAlater®), and preservation time period (4 weeks or 12 weeks). [file 12936_2021_3754_MOESM5_ESM.pdf]

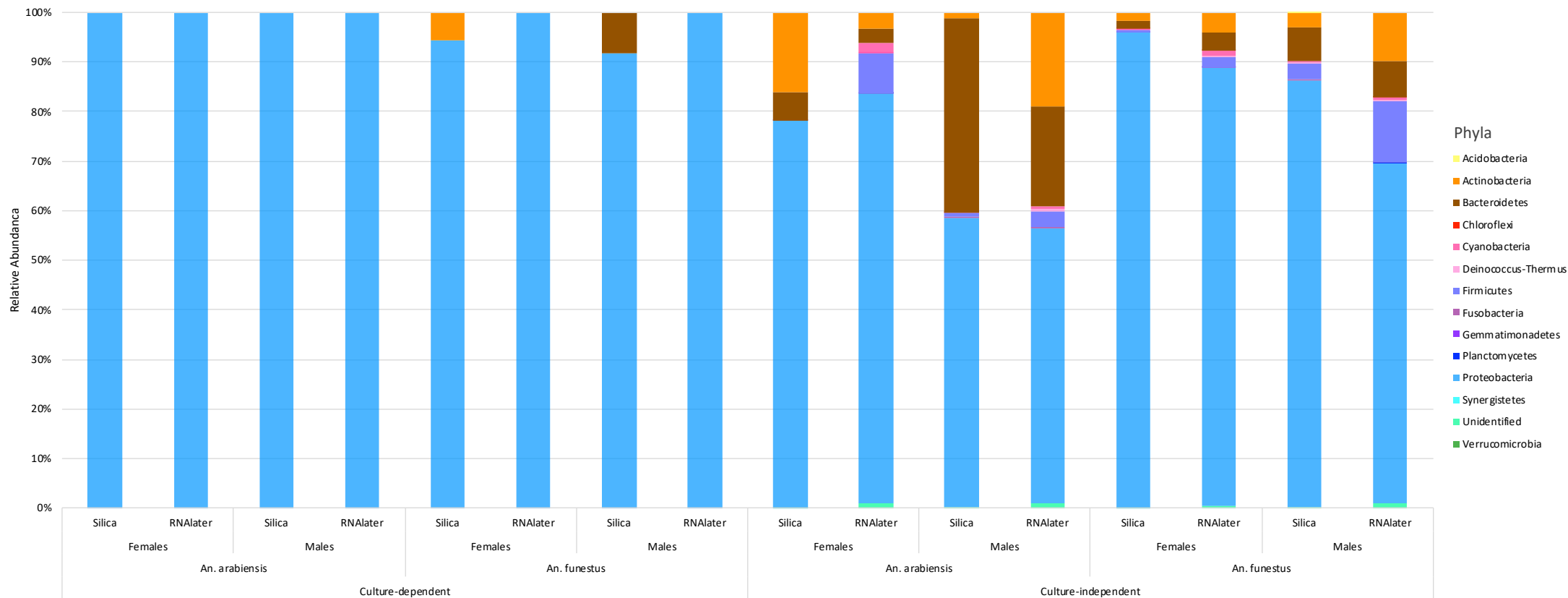

Supplement: Supplementary file 11 — Additional file 11. Accumulative bacterial phyla identified by culture-dependent and culture-independent techniques from preserved mosquitoes. Phyla are characterized according to species (An. arabiensis or An. funestus), sex (female or male), and preservative (silica or in RNAlater®). [file 12936_2021_3754_MOESM11_ESM.pdf]

A

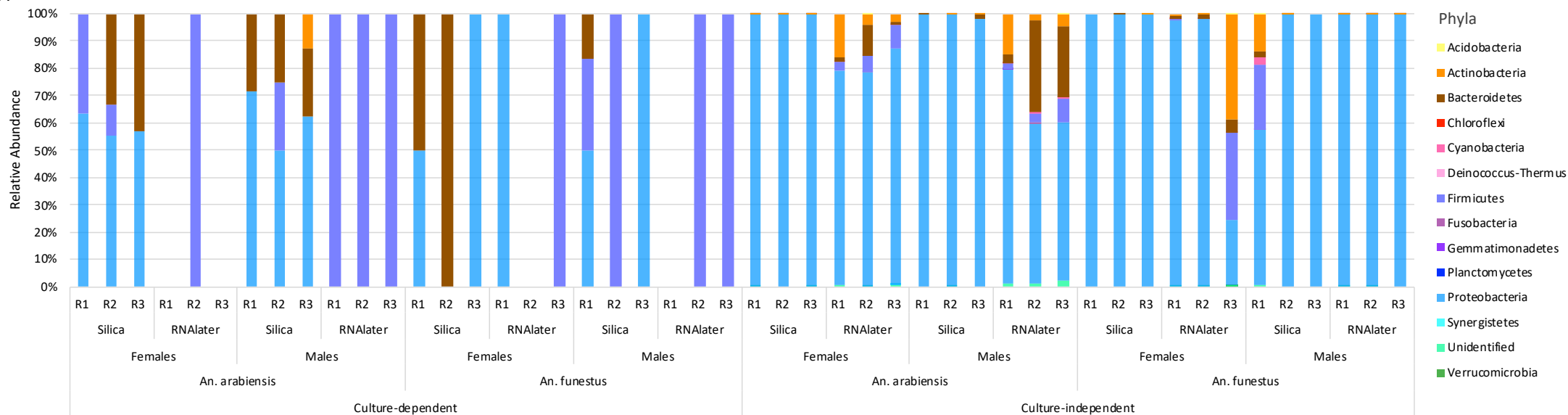

B

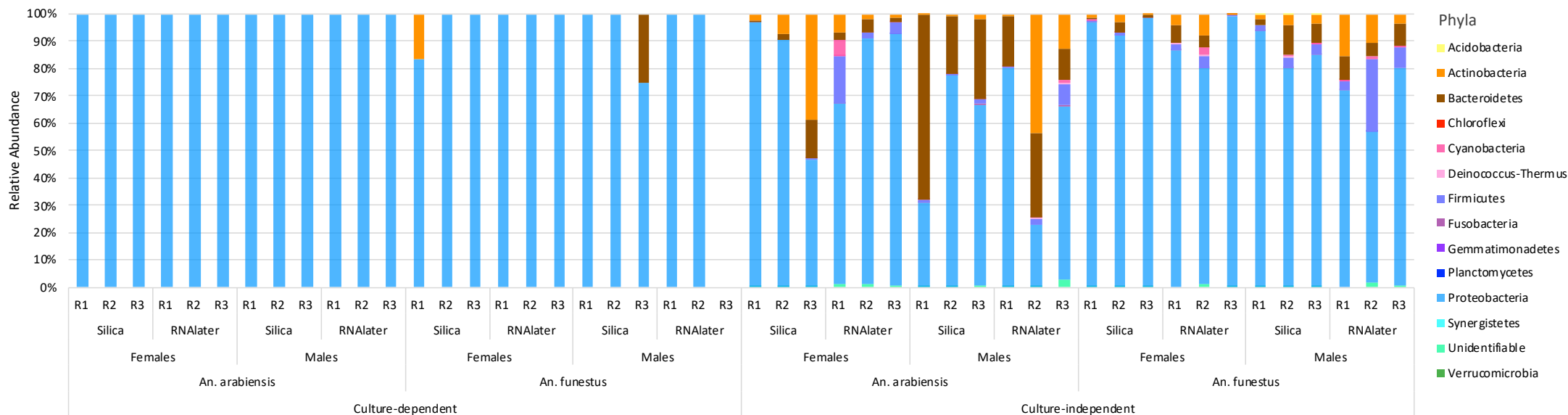

Supplement: Supplementary file 12 — Additional file 12. Bacterial phyla identified from mosquitoes preserved for (A) 4 weeks and (B) 12 weeks. Phyla are characterized according to technique (culture-dependent or culture-independent), species (An. arabiensis or An. funestus), sex (female or male), and preservative (silica or in RNAlater®). Phyla are recorded for all replicates (R). [file 12936_2021_3754_MOESM12_ESM.pdf]

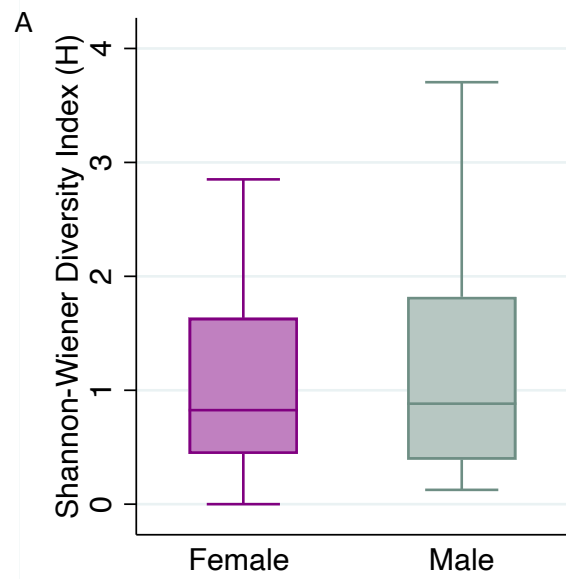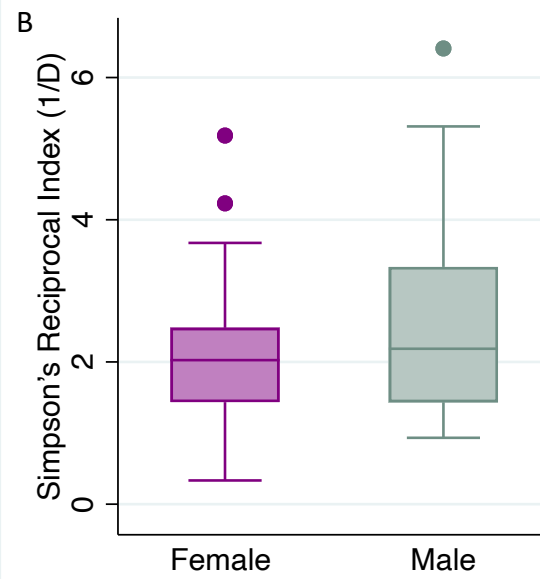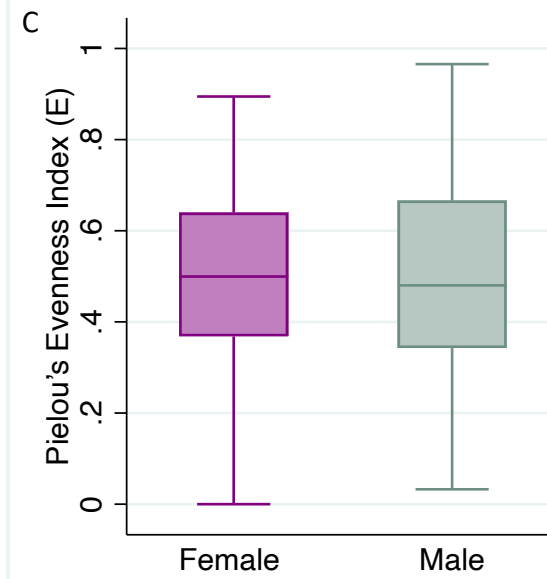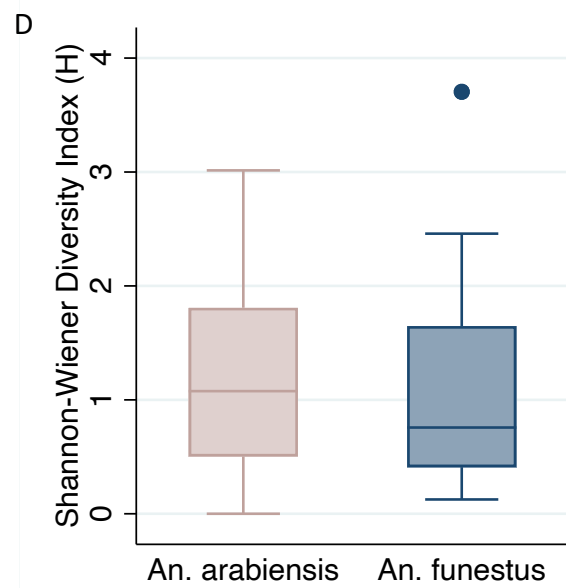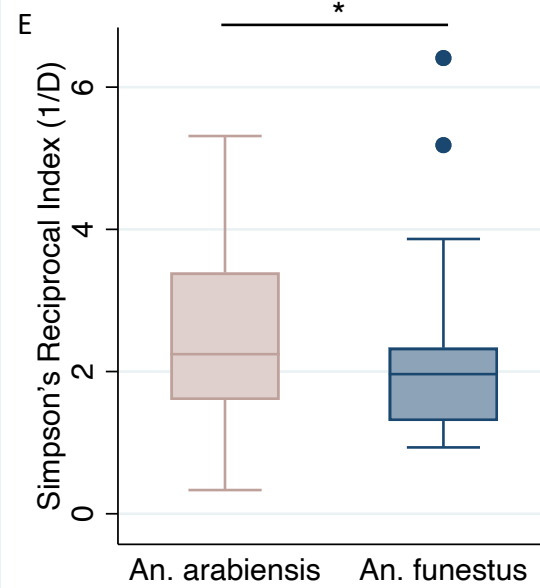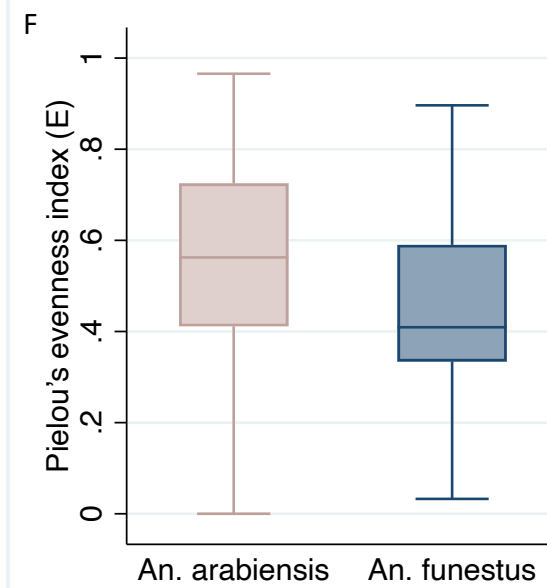

Supplement: Supplementary file 13 — Additional file 13. Box plots of diversity indices calculated for (A, B, C) sex and (D, E, F) species. Upper and lower limits of boxes represent quartiles around the mean and horizontal lines within boxes represent median values for (A, D) Shannon-Wiener (H), (B, E) Simpson’s reciprocal (1/D), and (C, F) Pielou’s evenness (E). Significant differences were calculated with Wilcoxon rank-sum (Mann-Whitney) tests (*P<0.05). [file 12936_2021_3754_MOESM13_ESM.pdf]

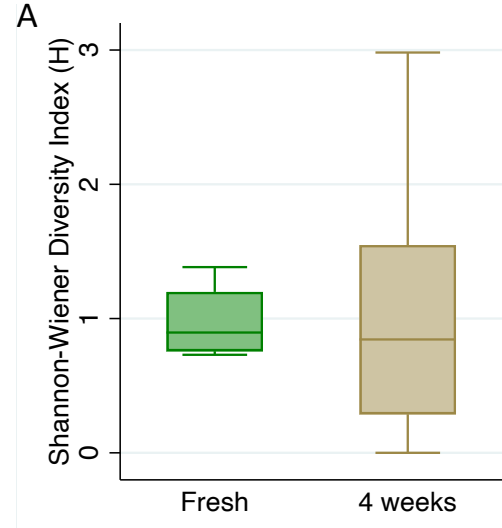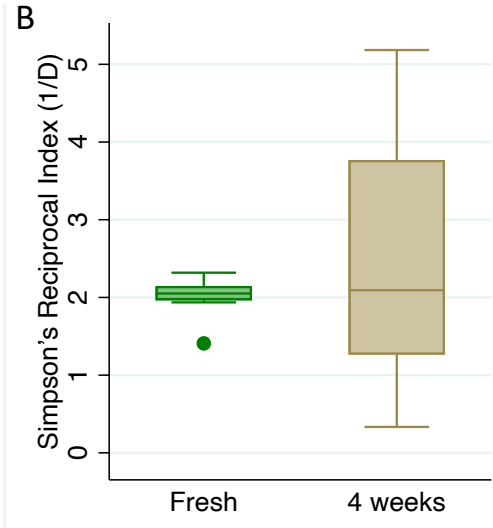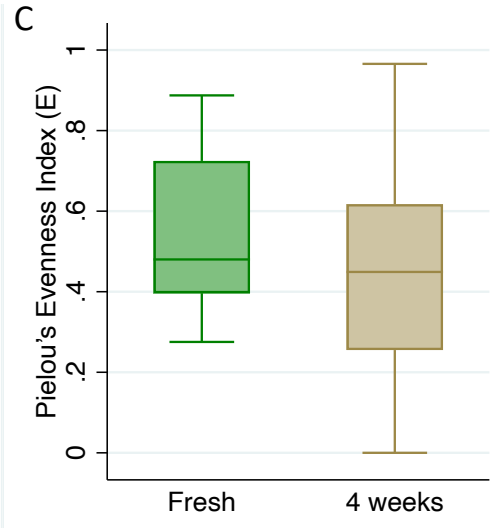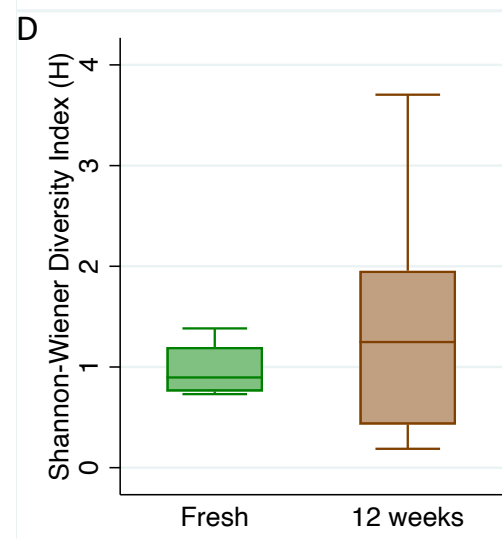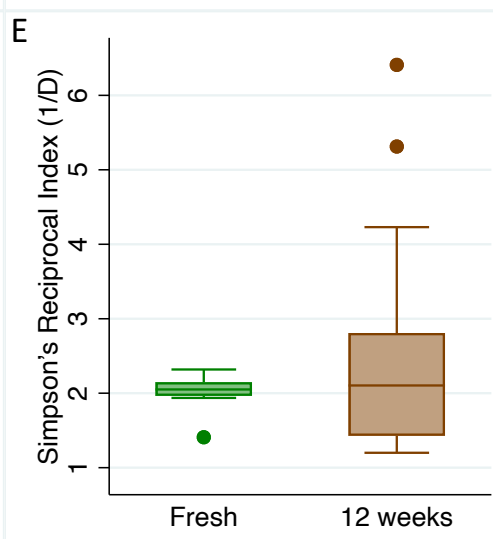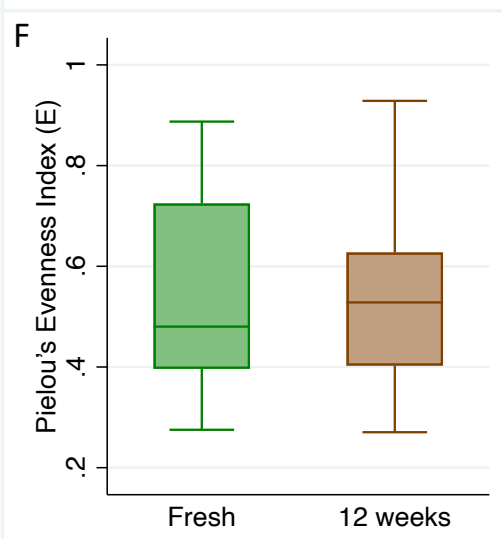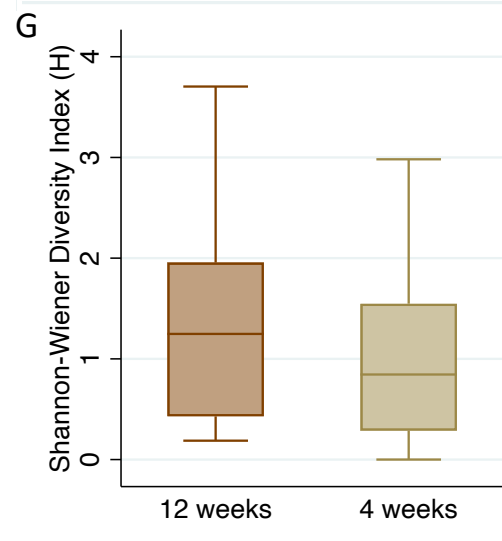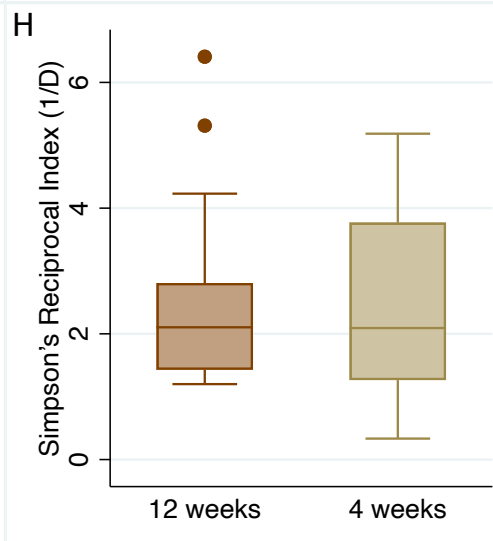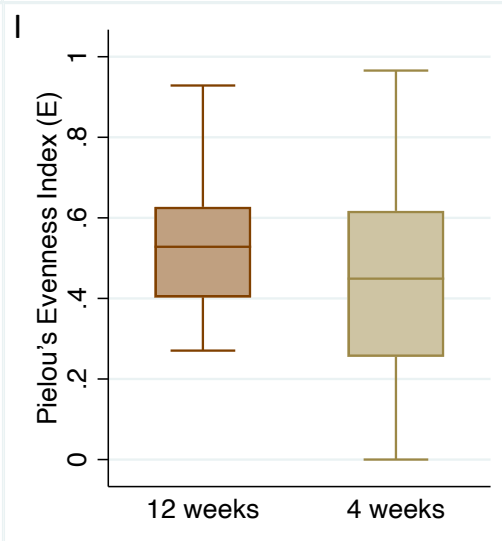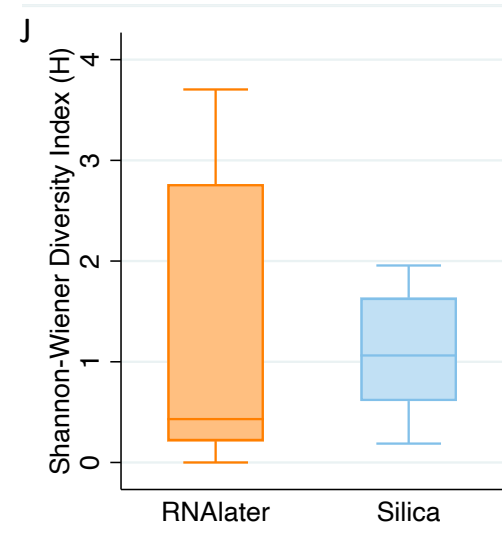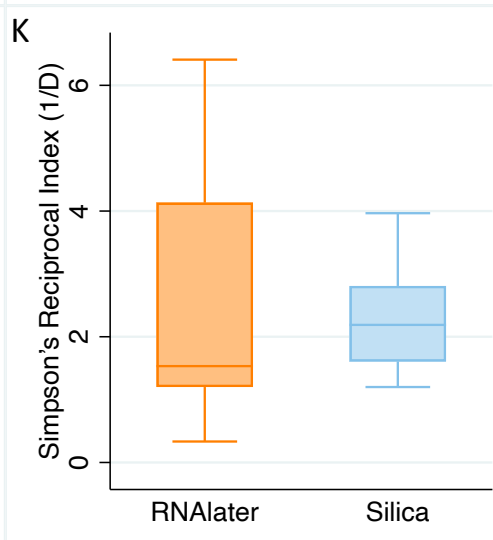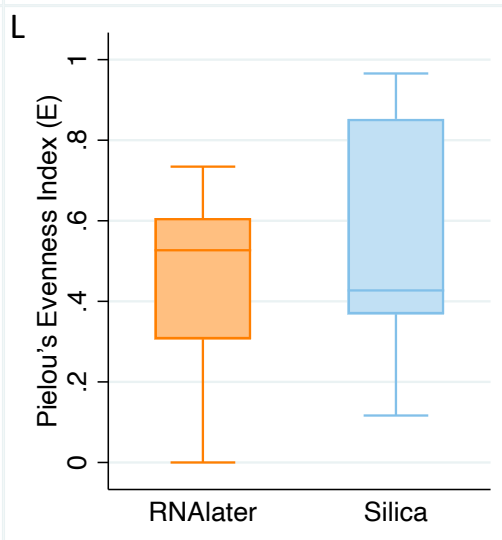

Supplement: Supplementary file 14 — Additional file 14. Box plots of diversity indices comparing (A, B, C) fresh mosquitoes and mosquitoes preserved for 4 weeks (D, E, F) fresh mosquitoes and mosquitoes preserved for 12 weeks, (G, H, I) mosquitoes preserved for 4 weeks and mosquitoes preserved for 12 weeks, (J, K, L) silica- and RNAlater®-preserved mosquitoes. Upper and lower limits of boxes represent quartiles around the mean and horizontal lines within boxes represent median values for (A, D, G, J) Shannon-Wiener (H), (B, E, H, K) Simpson’s reciprocal (1/D), and (C, F, I, L) Pielou’s evenness (E). Significant differences were calculated with Wilcoxon rank-sum (Mann-Whitney) tests. [file 12936_2021_3754_MOESM14_ESM.pdf]
