# Supplementary material for: Microbiota identified from preserved Anopheles
Source: Malar J. 2021 May 22;20:230. doi: 10.1186/s12936-021-03754-7 (PMC8141131; doi:10.1186/s12936-021-03754-7)
Supplement: Supplementary file 8 — Additional file 8. Bacteria identified by (A, B, C) culture-dependent and (D, E, F) culture-independent techniques from Anopheles funestus. Bacteria were identified from fresh (A, D) females, (B, E) males, and (C, F) the larval rearing water. Bacteria indistinguishable by MALDI-TOF MS include 1A. hydrophila and A. veronii; 2C. propinquum and C. pseudodiphtheriticum; 3D. acidovorans and D. tsuruhatensis; 4E. anophelis, E. meningoseptica, and E. miricola; 5K. oxytoca, R. ornithinolytica, R. planticola, and R. terrigena; and 6S. fonticola and S. oryzae. [file 12936_2021_3754_MOESM8_ESM.pdf]

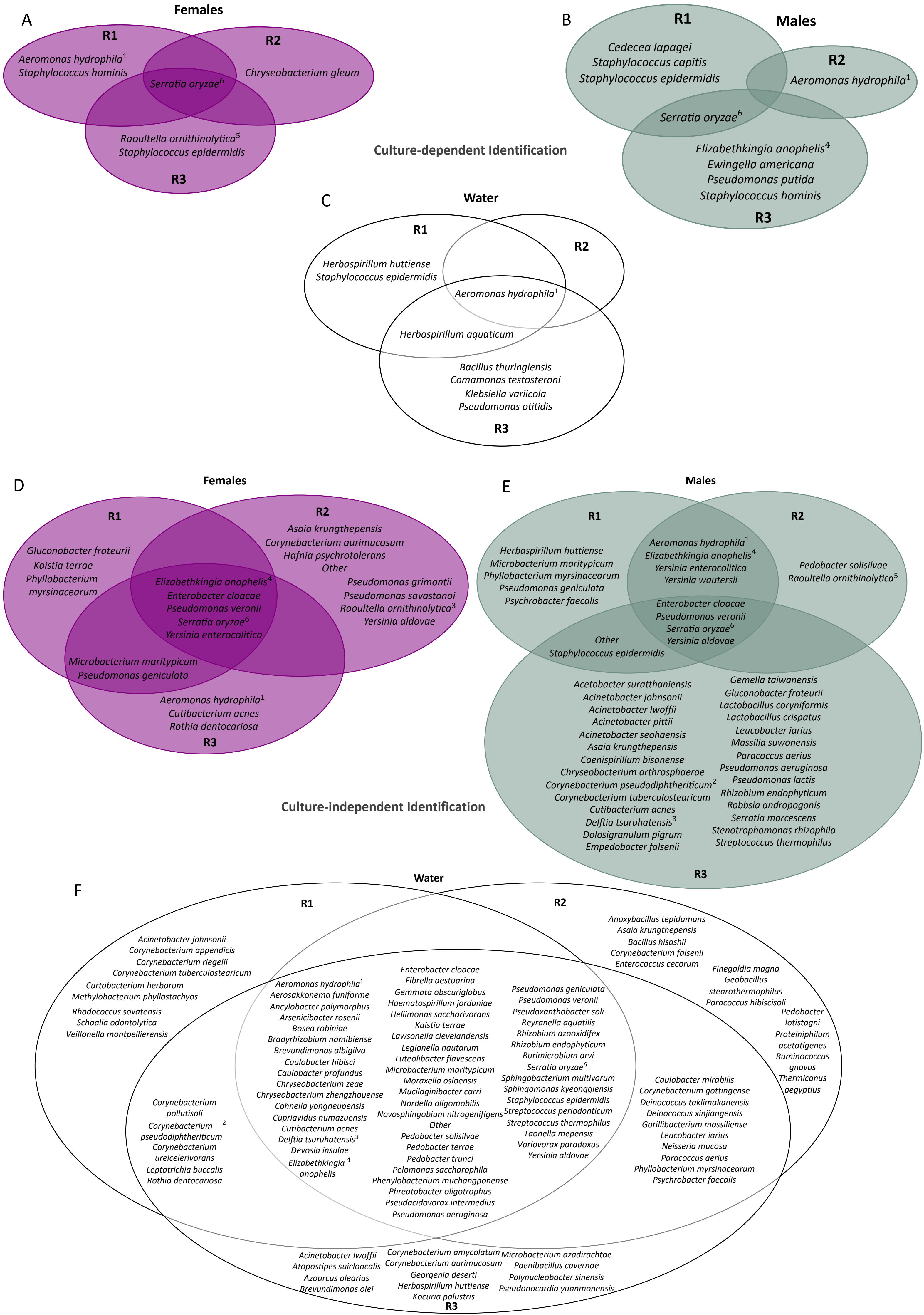
A
Females
R1
Aeromonas hydrophila[1]
Staphylococcus hominis
R2
Chryseobacterium gleum
Serratia oryzae[6]
Raoultella ornithinolytica[5]
Staphylococcus epidermidis
R3
B
Males
R1
Cedecea lapagei
Staphylococcus capitis
Staphylococcus epidermidis
R2
Aeromonas hydrophila[1]
Serratia oryzae[6]
Elizabethkingia anophelis[4]
Ewingella americana
Pseudomonas putida
Staphylococcus hominis
R3
Culture-dependent Identification
C
Water
R1
Herbaspirillum huttiense
Staphylococcus epidermidis
R2
Aeromonas hydrophila[1]
Herbaspirillum aquaticum
Bacillus thuringiensis
Comamonas testosteroni
Klebsiella variicola
Pseudomonas otitidis
R3
D
Females
R1
Gluconobacter frateurii
Kaistia terrae
Phyllobacterium myrsinacearum
R2
Asaia krungthepensis
Corynebacterium aurimucosum
Hafnia psychrotolerans
Other
Pseudomonas grimontii
Pseudomonas savastanoi
Raoultella ornithinolytica[3]
Yersinia aldovae
Elizabethkingia anophelis[4]
Enterobacter cloacae
Pseudomonas veronii
Serratia oryzae[6]
Yersinia enterocolitica
Microbacterium maritypicum
Pseudomonas geniculata
Aeromonas hydrophila[1]
Cutibacterium acnes
Rothia dentocariosa
R3
E
Males
R1
Herbaspirillum huttiense
Microbacterium maritypicum
Phyllobacterium myrsinacearum
Pseudomonas geniculata
Psychrobacter faecalis
R2
Aeromonas hydrophila[1]
Elizabethkingia anophelis[4]
Yersinia enterocolitica
Yersinia wautersii
Pedobacter solisilvae
Raoultella ornithinolytica[5]
Enterobacter cloacae
Pseudomonas veronii
Serratia oryzae[6]
Yersinia aldovae
Other
Staphylococcus epidermidis
Acetobacter suratthaniensis
Acinetobacter johnsonii
Acinetobacter lwoffii
Acinetobacter pittii
Acinetobacter seohaensis
Asaia krungthepensis
Caenispirillum bisanense
Chryseobacterium arthrosphaerae
Corynebacterium pseudodiphtheriticum[2]
Corynebacterium tuberculostearicum
Cutibacterium acnes
Delftia tsuruhatensis[3]
Dolosigranulum pigrum
Empedobacter falsenii
Gemella taiwanensis
Gluconobacter frateurii
Lactobacillus coryniformis
Lactobacillus crispatus
Leucobacter iarius
Massilia suwonensis
Paracoccus aerius
Pseudomonas aeruginosa
Pseudomonas lactis
Rhizobium endophyticum
Robbsia andropogonis
Serratia marcescens
Stenotrophomonas rhizophila
Streptococcus thermophilus
R3
Culture-independent Identification
F
Water
R1
R2
Acinetobacter johnsonii
Corynebacterium appendicis
Corynebacterium riegelii
Corynebacterium tuberculostearicum
Curtobacterium herbarum
Methylobacterium phyllostachyos
Rhodococcus sovatensis
Schaalia odontolytica
Veillonella montpellierensis
Anoxybacillus tepidamans
Asaia krungthepensis
Bacillus hisashii
Corynebacterium falsenii
Enterococcus cecorum
Finegoldia magna
Geobacillus stearothermophilus
Paracoccus hibiscisoli
Pedobacter lotistagni
Proteiniphilum acetatigenes
Ruminococcus gnavus
Thermicanus aegyptius
Aeromonas hydrophila[1]
Aerosakkonema funiforme
Ancylobacter polymorphus
Arsenicibacter rosenii
Bosea robiniae
Bradyrhizobium namibiense
Brevundimonas albigilva
Caulobacter hibisci
Caulobacter profundus
Chryseobacterium zeae
Chryseobacterium zhengzhouense
Cohnella yongneupensis
Cupriavidus numazuensis
Cutibacterium acnes
Delftia tsuruhatensis[3]
Devosia insulae
Elizabethkingia anophelis[4]
Enterobacter cloacae
Fibrella aestuarina
Gemmata obscuriglobus
Haematospirillum jordaniae
Heliimonas saccharivorans
Kaistia terrae
Lawsonella clevelandensis
Legionella nautarum
Luteolibacter flavescens
Microbacterium maritypicum
Moraxella osloensis
Mucilaginibacter carri
Nordella oligomobilis
Novosphingobium nitrogenifigens
Other
Pedobacter solisilvae
Pedobacter terrae
Pedobacter trunci
Pelomonas saccharophila
Phenylobacterium muchangponense
Phreatobacter oligotrophus
Pseudacidovorax intermedius
Pseudomonas aeruginosa
Pseudomonas geniculata
Pseudomonas veronii
Pseudoxanthobacter soli
Reyranella aquatilis
Rhizobium azooxidifex
Rhizobium endophyticum
Rurimicrobium arvi
Serratia oryzae[6]
Sphingobacterium multivorum
Sphingomonas kyeonggiensis
Staphylococcus epidermidis
Streptococcus periodonticum
Streptococcus thermophilus
Taonella mepensis
Variovorax paradoxus
Yersinia aldovae
Corynebacterium pollutisoli
Corynebacterium pseudodiphtheriticum[2]
Corynebacterium ureicelerivorans
Leptotrichia buccalis
Rothia dentocariosa
Caulobacter mirabilis
Corynebacterium gottingense
Deinococcus taklimakanensis
Deinococcus xinjiangensis
Gorillibacterium massiliense
Leucobacter iarius
Neisseria mucosa
Paracoccus aerius
Phyllobacterium myrsinacearum
Psychrobacter faecalis
Acinetobacter lwoffii
Atopostipes suicloacalis
Azoarcus olearius
Brevundimonas olei
Corynebacterium amycolatum
Corynebacterium aurimucosum
Georgenia deserti
Herbaspirillum huttiense
Kocuria palustris
Microbacterium azadirachtae
Paenibacillus cavernae
Polynucleobacter sinensis
Pseudonocardia yuanmonensis
R3
